# The adipose tissue-plaque crosstalk: omics profiling of perivascular adipose tissues for understanding plaque stability

**DOI:** 10.1186/s12933-026-03276-z

**Published:** 2026-07-06

**Authors:** Marija Tomašević, Sandra Vladimirov, Christos Tsatsanis, Eleni Paflioti, Dimitris Kardassis, Isidoros Axiotis, Despoina Lazaridou, Laura Comi, Claudia Giglione, Ana Ninić, Sanja Erceg, Tamara Ratković, Aleksandar Davidović, Marko Milanov, Luka Dodić, Gordana Mihajlović, Paolo Magni, Miron Sopić

**Affiliations:** 1 Department of Medical Biochemistry, Faculty of Pharmacy, University of Belgrade, Vojvode Stepe 450, Belgrade, Serbia; 2 Laboratory Clinical Chemistry, School of Medicine, University of Crete, Rethymno, Greece; 3 Institute of Molecular Biology and Biotechnology, Foundation for Research and Technology of Hellas, 71003 Heraklion, Greece; 4 Laboratory of Biochemistry, Division of Basic Medical Sciences, University of Crete Medical School, Heraklion, Greece; 5 Department of Pharmacological and Biomolecular Sciences “Rodolfo Paoletti”, Università Degli Studi Di Milano, Via Balzaretti 9, 20133 Milan, Italy; 6 Department for Internal Medicine, Faculty of Dentistry, University of Belgrade, Belgrade, Serbia; 7 Intern Clinic, Clinical Ward for Cardiovascular Diseases, Clinical-Hospital Centre Zvezdara, Belgrade, Serbia; 8 Faculty of Medicine, University of Belgrade, Belgrade, Serbia; 9 Department for Gerontology, Clinical-Hospital Centre Zvezdara, Belgrade, Serbia; 10 IRCCS MultiMedica, Via Milanese 300, 20099 Sesto San Giovanni, Milan, Italy

**Keywords:** Adipose tissue, Perivascular, Inflammation, Atherosclerotic plaque, Omics

## Abstract

Atherosclerotic cardiovascular disease (ASCVD) remains the leading cause of global morbidity and mortality, despite significant advances in lipid-lowering and cardiometabolic therapies. Increasing evidence indicates that atherosclerosis cannot be fully understood as a disease confined to the arterial wall, but rather reflects complex interactions between vascular, immune, metabolic, and perivascular compartments. Among these, perivascular adipose tissue (PVAT) has emerged as a critical regulator of vascular homeostasis and plaque biology due to its unique anatomical proximity and functional continuity with the vessel wall. This review summarises current knowledge on the role of PVAT in atherosclerosis, particularly insights gained through omics approaches. We explore how transcriptomics, proteomics, metabolomics, and lipidomics have advanced the structural and functional characterisation of PVAT, revealing pronounced cellular heterogeneity, depot-specific signatures, and dynamic phenotypic plasticity, including brown-to-white adipose tissue transitions. We further examine the molecular and cellular interfaces that mediate PVAT–vascular crosstalk and discuss how PVAT influences key processes relevant to plaque development and stability, including inflammation and immune signaling, extracellular matrix remodeling, cell differentiation and survival, and regulation of vascular tone. Importantly, emerging evidence supports bidirectional communication between PVAT and atherosclerotic plaques, establishing maladaptive feedback loops that may sustain vascular inflammation and contribute to residual cardiovascular risk. Finally, we address translational implications, evaluating the potential of PVAT-derived biomarkers and the therapeutic targeting of PVAT–plaque interaction for improved risk stratification and the development of more precise, tissue-targeted interventions in ASCVD.

**Graphical abstract:**

**Figure:**
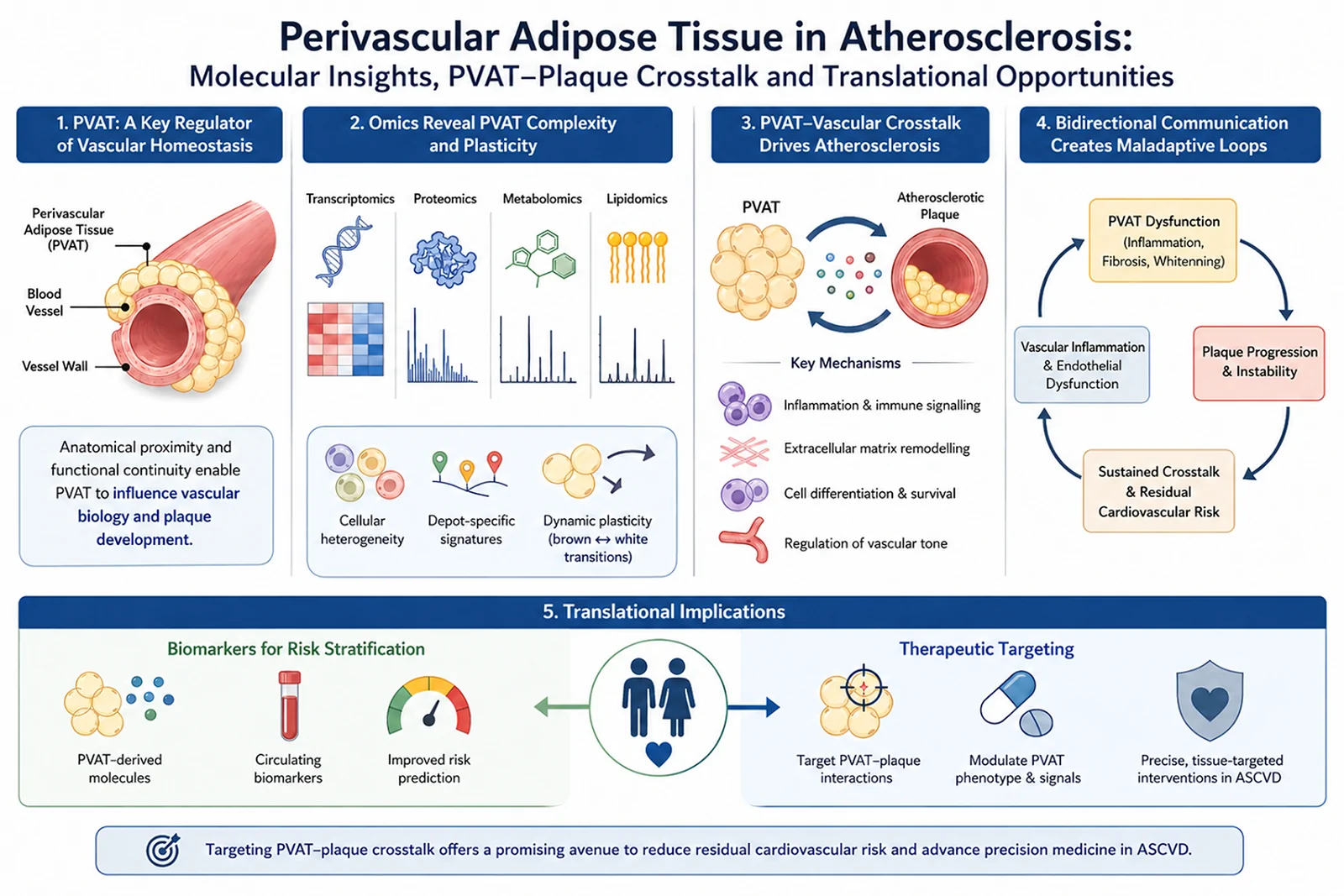

## Research insights

**What is already known about this topic?**

Atherosclerotic cardiovascular disease (ASCVD) remains the leading cause of morbidity and mortality. Despite optimal lipid-lowering therapy, substantial residual cardiovascular risk persists, driven in part by unresolved vascular inflammation. Perivascular adipose tissue (PVAT) has emerged as a metabolically active and immunologically responsive regulator of vascular function, but its role in plaque biology is not fully defined.

**What is the key research question?**

How does PVAT contribute to plaque development and instability, and can omics-derived signatures of PVAT improve cardiovascular risk stratification and therapeutic targeting?

**What is new?**

This review integrates evidence from different omics to characterize PVAT as a heterogeneous, dynamic tissue. It highlights bidirectional PVAT–plaque crosstalk mediated by adipokines, lipid mediators, extracellular vesicles, and non-coding RNAs as a key driver of vascular inflammation, matrix remodeling, and plaque instability, positioning PVAT as a major contributor to residual cardiovascular risk.

**How might this study impact clinical practice?**

PVAT imaging and molecular profiling may enhance detection of biologically active disease and refine risk stratification. Targeting PVAT–vascular interactions represents a promising strategy for reducing residual inflammatory risk and enabling more precise, tissue-specific therapies in high-risk patients.

## Introduction

Atherosclerotic cardiovascular disease (ASCVD) affects over 400 million people worldwide and was responsible for nearly 19 million deaths in 2023 [1], remaining the leading cause of morbidity and mortality. Its pathogenesis involves a complex interplay of cellular and molecular mechanisms that drive the formation, progression, and destabilisation of atherosclerotic plaques. Traditionally, atherosclerotic plaques have been classified based on their morphological features into two broad phenotypes: unstable and stable plaques. [2]. However, despite growing recognition that plaque phenotypes show substantial biological heterogeneity and different therapeutic responses, current approaches to risk stratification, diagnosis, and clinical classification still rely mainly on anatomical and clinical features, with limited consideration of plaque-intrinsic biology or the contribution of surrounding vascular and perivascular tissues. While lipid lowering, blood pressure control, and tobacco cessation have reduced the prevalence of classical high-risk plaque features, these advances have coincided with a global rise in obesity and metabolic disorders, including metabolic syndrome, insulin resistance, and type 2 diabetes mellitus, which are likely to reshape plaque composition, inflammatory activity, and residual cardiovascular risk [3–6]. This residual risk is increasingly being attributed to chronic inflammation within the vascular wall, which promotes plaque progression toward more vulnerable phenotypes and sustains disease activity over time. Clinical trials targeting inflammation independently of low-density lipoprotein cholesterol (LDL-C) reduction, using interleukin-1β monoclonal antibodies [7] or low dose colchicine [8, 9], have demonstrated meaningful reductions in major adverse cardiovascular events (MACE). Furthermore, recent FDA approval of colchicine as first anti-inflammatory therapy for ASCVD complication risk management paves the way for further investigation into immune system modulation as a therapeutic strategy [10]. Despite these efforts, therapeutics capable of specifically targeting vascular inflammation remain limited, while most available anti-inflammatory agents act systemically and carry the risk of clinically relevant adverse effects. The development of more selective, plaque-directed anti-inflammatory approaches therefore represents a critical unmet need. Additionally, increasing evidence indicates that atherosclerosis cannot be fully understood as a disease confined to the arterial wall alone. Adipose tissue has emerged as a dynamic biological compartment in which disturbances in lipid handling and inflammation intersect, and in recent years its influence on cardiovascular health has gained significant attention. Among adipose depots, particular focus has been placed on perivascular adipose tissue (PVAT), which surrounds most blood vessels and is in direct anatomical continuity with the vascular wall. Far from being a passive fat layer, PVAT is a metabolically active and immunologically responsive organ that communicates with the vasculature via paracrine mediators, extracellular vesicles, and direct cell–cell interactions, thereby shaping vascular tone, endothelial function, and atherosclerotic plaque development. Because of this close spatial relationship, alterations in PVAT physiology directly impact the function of surrounding organs and structures. In the setting of metabolic dysregulation, PVAT undergoes a pro-inflammatory shift, with changes in cellular composition and secretory profile creating a paracrine influence that negatively affects the local microenvironment, including the vascular wall [11, 12].

Collectively, these observations highlight the need for novel conceptual and methodological approaches able to capture the molecular, cellular, and metabolic complexity of atherosclerotic plaques and their interactions with the surrounding PVAT. In this context, omics-based approaches provide unprecedented opportunities to resolve plaque heterogeneity, uncover previously unrecognised disease pathways, and guide more precise strategies for the diagnosis, prognosis, and treatment of ASCVD [13].

Therefore, in this review, we focus on molecular pathways mediating PVAT–plaque crosstalk, as revealed by omics studies, and highlight their potential to uncover novel mechanistic targets, biomarkers, and therapeutic strategies for modulating the perivascular microenvironment to enhance risk stratification and clinical management of ASCVD.

## ASCVD-relevant properties of perivascular adipose tissue

PVAT is defined as the layer of adipose tissue located within a distance equal to the luminal diameter of the artery. For larger arteries such as the aorta, PVAT extends up to 2 cm from the outer surface of the vessel [14]. Depending on the localisation, different PVAT depots show variation in phenotype and function, cell composition and stage of differentiation from progenitor cells, and are classified as thoracic (tPVAT), abdominal (aPVAT), coronary (cPVAT) and mesenteric (mPVAT) [15]. Adipose tissue can be morphologically classified into white (WAT), brown (BAT), and beige types. Aortic PVAT can be subdivided into thoracic PVAT (tPVAT) and abdominal PVAT (aPVAT), reflecting distinct anatomical and functional characteristics along the aorta. While tPVAT consists primarily of brown adipocytes with multilocular lipid droplets, aPVAT comprises a mixture of brown and white adipocytes and shows lower levels of BAT markers compared with tPVAT [16]. Despite containing mostly white adipocytes with unilocular and small lipid droplets, cPVAT exhibits a beige adipose tissue transcriptional signature [17], while mPVAT consists predominantly of white adipocytes with large unilocular lipid droplets and low mitochondrial density [18]. PVAT was originally thought to confer exclusively structural support to the vessels but this notion was later abandoned. The type of adipose tissue largely determines its effects on the vascular wall and distinct AT depots generate different types and quantities of adipokines and cytokines, enabling communications among adipocytes, immune cells, vascular smooth muscle cells (VSMCs), and endothelial cells (ECs) in vascular microenvironment [19, 20]. This, “outside to inside’’ signaling direction was extensively discussed, but new evaluation led to the proposition of a bi-directional crosstalk, as the signals from inside the vascular lumen can impact the overlaying tissue (inside-out mechanism) [21–23]. In physiological conditions, this crosstalk reflects anti-atherogenic properties of PVAT, such as thermogenesis and regulation of fatty acid metabolism, but in pathological conditions (mainly obesity), this depot undergoes a pro-inflammatory shift [24–26]. Pathological changes related with dysfunctional PVAT include alteration in the cellular composition, particularly adipocyte hypertrophy and loss of thermogenic characteristics, increased infiltration of macrophages and other immune cells, and expansion or activation of stromal/fibroblast-like cells. These changes are accompanied by whitening, decreased fatty acid oxidation, increased production of reactive oxygen species (ROS), and increased energy storage [12, 27]. Changes also lead to increased production of inflammatory adipokines and cytokines, such as tumor necrosis factor (TNF), IL-6, IL-1b, resistin, monocyte chemoattractant protein 1 (MCP-1), leptin and decreased production of adiponectin [11, 28–30]. These pro-atherogenic stimuli lead ECs to become dysfunctional, and SMCs to start migrating and proliferating [26, 31, 32]. All together, these factors significantly contribute to the progression of atherosclerosis [24]. In accordance with this, PVAT shows increased volume and inflammation in regions overlaying larger plaques, suggesting that atherogenic molecular signals from the plaque microenvironment alter local adipocyte function, highlighting the bidirectional crosstalk between vascular lesions and surrounding adipose tissue [33]. In the next section we focus more on the mechanisms that underlie the crosstalk between these two entities.

## Omics-based structural and functional characterization of PVAT

Recent advances in omics technologies, including genomics, transcriptomics, proteomics, and metabolomics, have provided insights into the molecular and cellular complexity of PVAT in health and disease [34–37]. Each of these omics layers provides complementary information. Genomics helps identify inherited or regulatory variants that may influence adipose tissue distribution, PVAT phenotype, and susceptibility to cardiometabolic dysfunction [38]. Transcriptomics, particularly single-cell and single-nucleus approaches, resolves the cellular composition of PVAT with high resolution and defines disease-associated transcriptional states in adipocytes, immune cells, endothelial cells, smooth muscle cells and stromal progenitors [39–41]. Proteomics captures the secreted and extracellular-matrix-associated proteins through which PVAT communicates with the vascular wall [42, 43], while metabolomics reveal bioactive metabolites, lipid mediators and energy pathways that regulate inflammation, oxidative stress, vascular tone and plaque remodeling [36, 41]. Together, these technologies provide a multidimensional framework for linking PVAT structure, cellular heterogeneity and molecular function to vascular pathology.

Single-cell approaches have identified discrete cell populations present in PVAT, defined their transcriptional states, and shown how these states shift in response to metabolic stress and inflammatory signals [41]. When combined with proteomic and metabolomic profiling, these data bring promise to resolve intercellular communication networks within PVAT and between PVAT and the vascular wall, capturing paracrine signaling via cytokines, adipokines, chemokines, metabolites, lipid mediators and extracellular vesicles [34–36, 42, 43]. To date, omics studies have demonstrated that PVAT is neither uniform nor passive: it comprises multiple cell types with distinct functional programmes and exhibits dynamic changes in response to both systemic and local conditions. Molecular signatures derived from these approaches clarify the mechanisms by which PVAT influences vascular homeostasis, modulates inflammatory pathways, and contributes to plaque development. This provides an empirical basis for understanding how PVAT participates in cardiovascular physiology and disease, and for identifying candidate biomarkers and intervention targets.

The following sections examine specific aspects of PVAT biology in the context of atherosclerotic cardiovascular disease, focusing on cellular heterogeneity, adipose phenotypic plasticity, PVAT–vascular interfaces, and the functional impact of PVAT changes on vascular and plaque biology.

## Cellular heterogeneity of PVAT

The cells present in PVAT can be classified as adipocytes and cells of the stromal vascular fraction (SVF). Adipocyte progenitor cells (APCs) are present in all PVAT depots, but their abundance and subtype composition vary markedly with anatomical location, age, and sex. Among others, cPVAT contains significantly fewer APCs and shows a more fibrotic, less adipogenic profile compared with mesenteric and abdominal PVAT, whereas mPVAT harbors the highest APC abundance with a progenitor population closely associated with blood vessels [38]. Lineage tracing and scRNA-seq in mice were utilized to define the cellular origins of PVAT adipocytes, showing that, during early life, they are derived from *Pdgfra* + *Pi16* + *Ly6a* + fibroblastic progenitors, which first give rise to *Pparg* + preadipocytes. However, in adult animals, classical preadipocytes are mostly replaced by a distinct population of *Pparg* + *Lpl* + *Fabp4* + ,,SMC-like’’ cells that share markers of SMCs and adipocytes, indicating a unique adipogenic route not observed in other fat depots [39]. Single-nucleus RNA-seq of human PVAT confirms a similar organization, identifying Uncoupling protein 1 + (*UCP1*) thermogenic adipocytes, fibroblastic progenitors, and *PPARG* + smooth-muscle-like precursor cells [39]. However, while mice possess *Myh11* + adipogenic SMCs, human PVAT harbors *PPARγ* + SMC-like cells that share smooth-muscle–associated pathways but lack *MYH11*, suggesting similar adipogenic roles but different levels of smooth-muscle identity. This supports the idea that PVAT is developmentally linked to the vascular wall and explains why PVAT can remodel in diseases affecting the vessel. In line with this developmental and phenotypic plasticity, a complementary study using scRNA-seq and functional assays also demonstrated that PVAT contains mesenchymal progenitors capable of differentiating towards SMCs [41]. Freshly isolated mouse PVAT stromal cells did not express mature SMC markers (*Acta2*, *Tagln*, *Cnn1*, *Myh11*); however, pseudotime trajectory analysis, which infers continuous cellular transitions from single-cell transcriptomic similarity [44], revealed a clear progression from *Ly6a* + fibroblast-like precursors to an *Acta2* + /*Myh11* + SMC state. In vitro, these PVAT-derived adipose stem cells readily adopted an SMC phenotype upon TGF-β1 stimulation and contributed to neointimal SMC accumulation in vivo after vascular injury [41]. These findings indicate that PVAT contains fibroblast-like precursors with the capacity to support vascular remodeling by supplying new SMCs, highlighting a bidirectional relationship between PVAT and the vessel wall.

Inflammatory cells, such as activated monocytes, macrophages and T-lymphocytes, are resident in PVAT [45] and they constitute around 2% of SVF cells in rodent PVAT in a healthy state [46], with significant accumulation in obesity-induced hypertension and atherosclerotic conditions [46, 47]. ScRNA sequencing of rat PVAT revealed a highly heterogeneous macrophage compartment composed of four transcriptionally distinct subsets. The *Gbp5* + macrophages were enriched in pro-inflammatory and pro-angiogenic programs, including *Il1b, Cxcl10/11, Ccl3, Ptgs2,* and VEGF-related pathways. In contrast, a unique *Pdpn* + macrophage subset, described to be PVAT specific, presented high expression of lipid-handling and cholesterol-efflux genes (*Apoe, Fabp4, Abca1, Pltp, Pparg1*) and insulin-responsive signatures, suggesting a homeostatic, anti-inflammatory phenotype [34]. T2DM increased the proportion of inflammatory *Gbp5* + macrophages and depleted the protective *Pdpn* + population not only in rat PVAT, but also in mouse and human PVAT obtained from the superior thyroid artery [34].

Similar studies were conducted using human samples. scRNA-seq of PVAT of patients with obstructive (OCA) and nonobstructive coronary atherosclerosis (NOCA), revealed diverse immune and stromal populations in healthy PVAT that shift markedly with atherosclerosis [35]. Diseased PVAT was characterized by a strong expansion of *SPP1* + inflammatory macrophages with lipid-handling signatures, *ACKR1* + endothelial cells that promote leukocyte recruitment, and *SOCS3* + smooth muscle cells exhibiting migratory and inflammatory activation. In parallel, profibrotic fibro-adipogenic progenitors (APOE^hi FAPs) increased in abundance, reflecting extensive extracellular matrix remodeling. Selected cell group populations present in PVAT local to atherosclerotic lesion are shown in Table 1.

**Table 1:** Cell clusters present in PVAT and their changes in patients in different stages of obstruction [35]

Cell cluster	Cell subcluster	Signature gene expression	Key pathways/functions (GO)	Differences across groups (NC → NOCA → OCA)
Macrophage cluster	Ma_1—Tissue-resident	LYVE1, MRC1, RNASE1, SELENOP	Anti-oxidant activity, tissue homeostasis	Present across all groups; no strong relation to disease
	Ma_2—Tissue-resident	LYVE1, MHC II,HLA-DPA1, HLA-DPB1, MHC II	Antigen presentation to CD4 T cells	Stable across groups; slight increase in atherosclerosis
	Ma_3—SPP1⁺ macrophages	SPP1, APOE, APOC1, FABP4, FABP5, LPL	ECM remodeling, fibrosis, chemotaxis, lipid handling	Most strongly increased in atherosclerosis; 5.42 × ↑ in NOCA and 4.04 × ↑ in OCA vs NC
	Ma_4—Classical monocyte-derived	FCN1, EREG, AREG, THBS1, VCAN	Acute inflammatory response, matrix remodeling	Increased modestly with disease—not dominant change
	Ma_5—Non-classical monocyte–derived	FCGR3A, SPN, SIGLEC10	Immune surveillance, patrolling behaviour	Present in all groups; no major differential enrichment
EC cluster (PECAM1)	EC0—Homeostatic / baseline ECs	RGCC, CA4, GPIHBP1, LPL, CD36	Baseline vascular homeostasis	Decreased with disease progression
	EC1- Inflammatory ACKR1⁺ ECs	PECAM1, ACKR1, POSTN	Leukocyte recruitment, endothelial activation, inflammation	Strongly expanded in atherosclerosis, especially OCA
	EC2 -Protective ECs	FTL, FTH1, TMSB4X	Iron availability, cell survival	Small population, relatively stable
	EC3—lymphatic ECs	PROX1, LYVE1, CCL21	Stress adaptation	Small population, relatively stable
	EC4—Arterial ECs	SOCS3, CNN1, ACTA2, TAGLN	flow-driven arterial remodeling and collateral arterial network development	Small population, relatively stable
FAP cluster (PDGFRA, CD34, CD59, or ITGB1)	FAP0—Profibrotic	APOE, PTN, COL15A1, and PTGDS	ECM organization, Profibrotic pathways	1.93- fold and 2.44-fold increase
	FAP1—General fibroblast	PLA2G2A and FN1		Decrease with disease
	FAP2—Adipose stem cells	CD55		No significant changes
	FAP3—Stressed FAP	JUN, FOS, JUNB, as well as HSP genes		No significant changes
	FAP4—Metabolically active	FABP4 and MT-ATP6		No significant changes
	FAP5	APOD		Small population
	FAP6	NRXN1		Small population

Together, these disease-associated macrophage, endothelial, SMC, and stromal populations form a pro-inflammatory, pro-fibrotic microenvironment in PVAT that closely tracks with coronary artery stenosis and supports plaque progression.

## Brown-to-white adipose phenotypic switching in PVAT

The type of adipose tissue is highly critical for its effect on the vascular wall. Because of its high thermogenic capacity, brown adipose tissue (BAT) markedly increases lipid and glucose utilization, thereby lowering circulating triglycerides and non-HDL cholesterol, which distinguishes it from white adipose tissue (WAT) and underlies its atheroprotective profile [48]. Omics analyses of murine and human PVAT reveal that this depot is a transcriptionally distinct adipose subtype with a strong thermogenic signature [39]. Bulk and single-cell transcriptomics demonstrated that tPVAT expresses high levels of classical BAT genes, including *Ucp1, Ppargc1a, Cycs, Cox5a, Cox7a2, and Elovl3*, supporting the concept that tPVAT is intrinsically programmed toward a “beige” (BAT-like) phenotype rather than resembling subcutaneous or visceral WAT [39]. Unlike tPVAT, aPVAT lacks these metabolic and paracrine features, with 4,834 DEGs between the two depots, leading to a markedly reduced thermogenic gene program and oxidation capacity [43], complementary with the fact that the abdominal aorta is more vulnerable to atherosclerosis than the thoracic aorta [49].

**Fig. 1:**
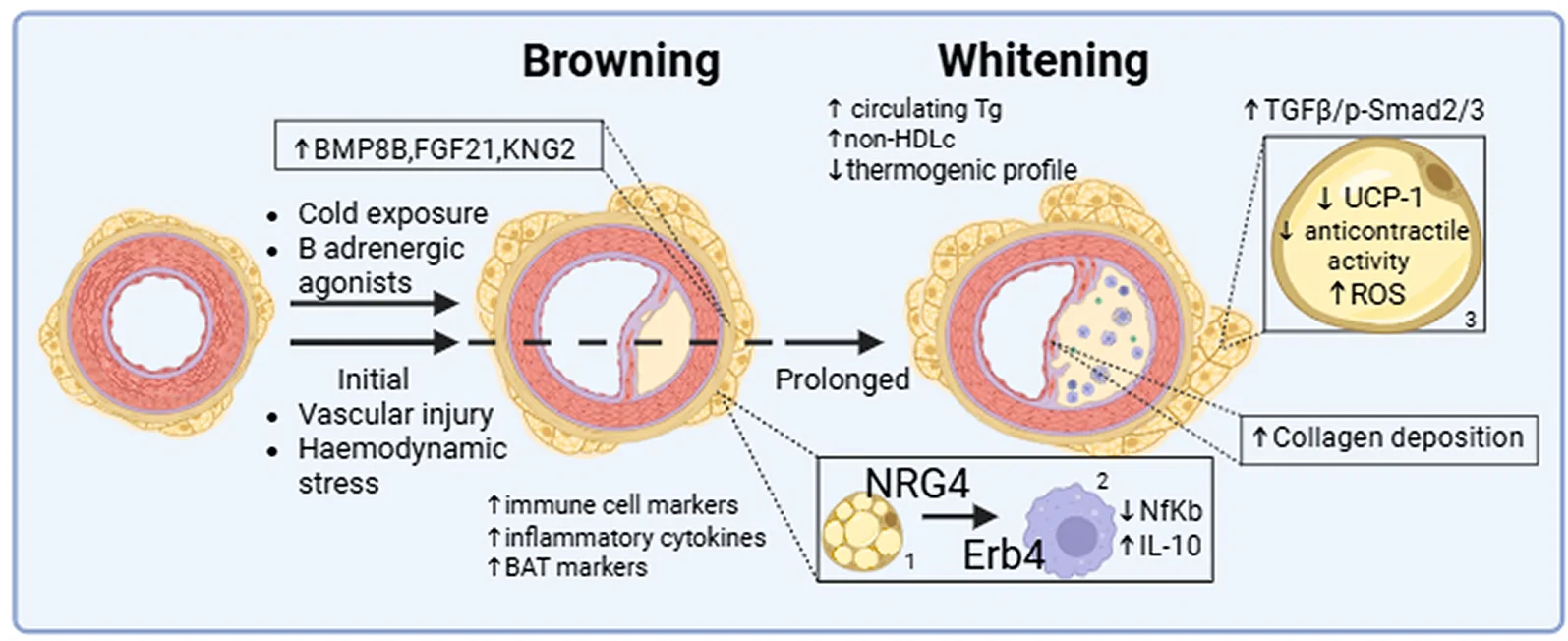
Signals governing phenotypic remodeling of perivascular adipose tissue (PVAT) toward a BAT-like (beige) or WAT-like (whitened) state and the resulting consequences for the vascular wall. In health, PVAT BAT-like phenotype is characterized by high mitochondrial content, UCP1 expression, and thermogenic capacity, contributing to lipid and glucose utilization and vascular protection [39]. Browning of PVAT can be induced by cold exposure, β-adrenergic signaling, or vascular stress and is associated with induction of BAT-related genes and BAT-okines (e.g. BMP8B, FGF21) [57], as well as NRG4-mediated macrophage polarization via ErbB4 signaling [58, 59]. In conditions that lead to vascular injury and haemodynamic stress browning is initially induced, but with prolonged exposure it leads to whitening [53, 55, 58]. In metabolic disease and advanced atherosclerosis, PVAT undergoes site-specific whitening marked by loss of thermogenic capacity, increased oxidative stress, TGF-β/Smad-dependent fibrosis, and reduced anticontractile activity, thereby promoting endothelial dysfunction and vascular inflammation [21, 53]. (1) Beige adipocyte; (2) ErbB4-expressing macrophage; (3) White adipocyte

The role of cPVAT and tPVAT is defined as protective in respect to vascular function, partly due to their character as BAT in a healthy state [16, 17, 50], but in metabolic diseases they undergo brown to white transition which is closely linked to the progression of CVD [51]. During early atherosclerosis, BAT features in tPVAT are preserved or even elevated due to the increased energy needs, but in advanced stages, site-specific whitening occurs only local to the plaques. This process is marked by increased collagen deposition and reduced UCP-1 expression, possibly as a result of the TGF-β/p-Smad2/3 signaling pathway [52]. As these changes and the elevated inflammation are shown to be present only above the plaque, these findings highlight the inside-out influence of endothelial lesion on PVAT function. In addition to stimulating local oxidative stress and inflammation, whitening of PVAT weakens its anti-contractile activity [18, 53]. Similarly, in hypertension animal models, vascular stress appears to initially induce browning in tPVAT, eventually leading to decline in BAT markers and vasodilatory function with disease progression, as shown in Fig. 1. This further supports the concept that intravascular signals, including hemodynamic stress, can dynamically modulate PVAT phenotype and function [54]. Animal studies have demonstrated that white-to-brown adipose tissue transition (beiging or browning) can be induced by interventions such as cold exposure or β3-adrenergic receptor agonists [55, 56]. These beneficial phenotypic shifts are accompanied by the upregulation of BAT-o-kines, such as bone morphogenetic protein-8b (BMP8B), fibroblast growth factor-21 (FGF21), and kininogen-2 (KNG2) [56]. Using bulk RNA sequencing of PVAT in a mouse model of endovascular injury [57], Adachi et al. identified a strong pro-inflammatory transcriptional activation characterized by upregulation of immune cell markers (*Cd11b, Cd11c, Mrc1, Adgre1*) and inflammatory cytokines (*Tnf, Serpine1, Il1a, Il1b*) in comparison to control. In parallel, the injury triggered a robust beige/brown adipocyte transcriptional program, with marked induction of BAT/beige markers such as *Ucp1, Cidea, Cox8b, Ppargc1a, Elovl3, Dio2*. Importantly, transcriptomic profiling revealed a substantial increase in Nrg4, a beige adipocyte–derived secretory factor, linking PVAT beigeing to macrophage polarization and inflammation resolution via the NRG4-ErbB4 pathway [57, 58]. Authors also suggested that these changes could be sex-dependant [57]. This mechanism showcases an important protective response in PVAT [57]. Inhibition of PVAT beigeing by genetically silencing PRDM16, a key regulator to beigeing, exacerbates inflammation and vascular remodeling following injury [57], underscoring the reparative and vasculoprotective potential of maintaining or restoring the brown adipose phenotype.

## Tissue interfaces of PVAT–vascular crosstalk

The crosstalk between PVAT and the underlying vessel wall is mediated through a diverse network of biochemical, mechanical, and cellular signals that collectively shape vascular homeostasis and disease. As it has already been described, PVAT regulates atherosclerotic plaque formation mostly due to its proximity to the vascular lumen.

One of the most important anatomical routes of PVAT-vascular communication is the vasa vasorum (VV), a microvascular network located in the adventitia that can expand towards the media and even neointima during disease [59]. Dysfunctional AT produces a range of pro-angiogenic and pro-inflammatory adipokines, including MCP-1, VEGF and TNF [60, 61], which can diffuse through the adventitia and directly interact with endothelial and smooth-muscle cells, promoting the formation of new vessels. In atherosclerotic plaques, neovascularization is the primary compensatory response to hypoxia and inflammatory conditions [62]. Evidence from fresh human cadaveric hearts demonstrates that lipid-rich plaques exhibit both a higher density of adventitial VV and increased expression of inflammatory and angiogenic factors in the adjacent epicardial adipose tissue, especially VEGFA and VEGFB [63]. In these conditions, VV can act as conduits for immune-cell trafficking [64], allowing macrophages to move between PVAT and the plaque, acquire oxidized LDL within the fat, and deliver it back into the intima [65].

Other than posing a means for communication, stability of the vasa vasorum is a significant factor for the stability of the overall lesion, as the intraplaque haemorrhage increases the vulnerability of the plaque [66, 67]. Adipose stem cells from PVAT are colocalized with VV and can interact with VSMCs via tissue factor signaling, supporting neovessel formation [37]. Adipose stem cells from ischemic heart cPVAT exhibit reduced tissue factor expression, impaired VSMC recruitment and angiogenic capacity, while restoring tissue factor levels rescues this defect. The weakening of this axis leads to the generation of structurally fragile, hyperpermeable microvessels prone to leakage and haemorrhage [37], directly influencing plaque stability.

Utilizing these structural routes of interaction, PVAT communicates with the vascular wall by direct release of mediators and through the release of extracellular vesicles, which serve as nanoscale carriers of lipids, proteins and regulatory RNAs that modulate endothelial and smooth muscle cell function [68]. Rab27a is shown to influence the contents of extracellular vesicles and their formation in ECs and adipose cells, mediating the endosome fusion with plasma membrane and the release of exosomes [69]. Genomic and proteomic profiling highlights RAB27A-dependent vesicular trafficking as a potentially relevant regulator of PVAT–vascular–cardiac crosstalk. Analyses utilizing human GWAS datasets revealed that both common and rare RAB27A variants are associated with BMI and glycemic traits, linking this gene to metabolic determinants of cardiovascular risk [42]. In Rab27a-null mouse models, SWATH-MS proteomics of PVAT, thoracic aorta, and cardiac tissue revealed coordinated dysregulation of multiple functional pathways [42]. PVAT and aortic proteomes from the male group showed marked downregulation of extracellular matrix proteins (COL1A1, COL1A2, COL3A1, FN1, LUM, DCN), consistent with phenotype annotations of altered aortic wall morphology. Concurrent reductions in contractile and cytoskeletal regulators (MYH6, ACTA1/ACTA2, TPM1, TNNT2, TNNI3, TTN) aligned with functional findings of enhanced α1-adrenergic vasoconstriction and reduced endothelium-dependent vasodilation. Broad suppression of mitochondrial oxidative phosphorylation proteins (NDUFA9, NDUFS2, UQCRC1/2, COX4I1, ATP5A1/ATP5B) pointed to impaired bioenergetics and changes in lipid-handling proteins (FABP4, ACADM, HADHA, CPT1B, PLIN1/2) indicated disrupted fatty-acid mobilization and storage within PVAT. These proteomic disturbances were observed predominantly in male PVAT and aorta, while females showed only minimal alterations [42]. PVAT-APC expression of Rab27a also regulates their differentiation capacity, and siRNA-mediated knockdown of Rab27a caused impaired adipogenesis and lipid mobilization [70].

## Functional roles of PVAT in vascular and plaque biology

### PVAT-mediated regulation of inflammation and immune signaling

Adipocytes share many common functions with innate immune cells, among which is production of inflammatory mediators and responses to inflammatory stimuli [71].

**Fig. 2:**
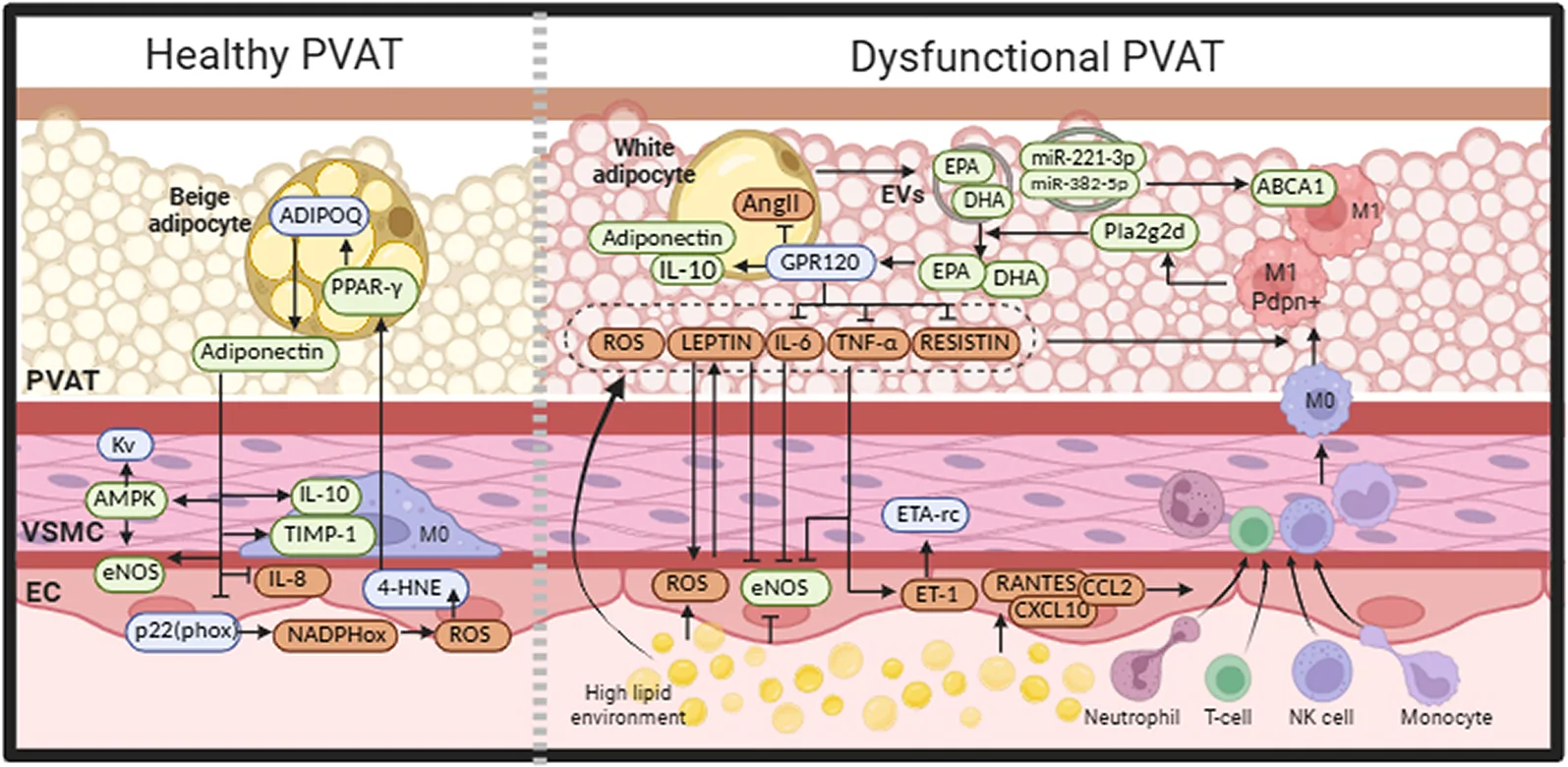
Healthy PVAT communicates with ECs, VSMCs, and immune cells through a balanced paracrine feedback loop characterized by PPAR-γ–dependent adiponectin production which counteracts the transient ROS and consequently maintains vascular homeostasis [73–75]. In contrast, dysfunctional PVAT, as observed in obesity and metabolic disease, undergoes a pro-inflammatory shift characterized by lipid influx, increased oxidative stress, hyperleptinemia, and elevated secretion of TNF, IL-6, resistin, angiotensin II, and pro-inflammatory lipid mediators [81, 82]. These factors converge on reduced endothelial nitric oxide synthase (eNOS) activity, enhanced endothelin-1 (ET-1) signaling, increased reactive oxygen species (ROS) production, and immune cell infiltration [83]. Accumulation of *Pdpn*^+^ macrophages expressing Pla2g2d represents a compensatory inflammation-resolving axis through the liberation of ω-3 fatty acids (DHA and EPA) from adipocyte-derived extracellular vesicles (EVs), leading to suppression of pro-inflammatory adipokines and angiotensin II signaling [37]. EVs also liberate micro-RNAs, among which miR-382-5p regulates macrophage lipid metabolism via ABCA1 and ABCG1 [84]. (AMPK, AMP-activated protein kinase; p22(phox), NADPH oxidase subunit p22phox; TIMP, tissue inhibitor of metalloproteinases; NADPHox, NADPH oxidase; ETA receptor, endothelin type A receptor; Ang II, angiotensin II; EPA/DHA, eicosapentaenoic and docosahexaenoic acid; RANTES, CCL5; CCL2, C–C motif chemokine ligand 2; CXCL10, C–X–C motif chemokine ligand 10, Kv—K^+^ voltage-gated channels))

Adiponectin is the most abundant adipokine produced by PVAT [51]. Adiponectin’s anti-atherogenic properties are crucial in preventing vascular disease progression, particularly in the context of obesity. It achieves this by inhibiting IL-8 expression in endothelial cells and boosting the production of anti-inflammatory IL-10 and tissue inhibitor of metalloproteinase-1 in macrophages [72]. Synthesis of adiponectin can be stimulated from inside the vascular lumen. In a healthy PVAT, transient increase of ROS and 4-hydroxynonenal (4-HNE) production in vascular wall stimulates the PPAR-γ mediated induction of adiponectin synthesis in neighbouring PVAT, consequently inhibiting arterial NADPH oxidase by downregulating p22(phox) through a phosphoinositide 3-kinase/Akt-mediated mechanism [73], as shown schematically in Fig. 2. The same pathway leads to elevated levels of NO by stimulating endothelial nitric-oxide synthetase (eNOS) and increasing the levels of tetrahydrobiopterin (BH4) [74]. This cycle reduces the oxidative damage in the microenvironment and highlights the importance of PVAT-vascular crosstalk, potentially emerging as an important therapeutic target for T2DM [73]. Additionally, adiponectin binds to Adipo1-receptors, widely expressed on VSMCs and ECs, and activates AMP-activated kinase (AMPK) leading to a vasodilatory effect by upregulation of potassium channels [75]. Activation of AMPK also causes the phosphorylation of eNOS and increased NO production [76, 77]. In hypertension and obesity-associated vascular dysfunction, reduced PVAT-derived adiponectin contributes to loss of PVAT anticontractile activity and impaired vascular homeostasis [78, 79], while elevated adiponectin levels in epicardial adipose tissue (EAT) enhance vascular function and decrease inflammation in coronary artery bypass patients [80], validating its cardioprotective effects.

PVAT responds to stimuli producing factors that act in an autocrine or paracrine fashion initiating signaling cascades that contribute to changes in the phenotype of cells. In accordance with this, endothelial cells exposure to a hyperlipidaemic environment drives lipid influx within the intima leading to increased production of cytokines and oxidative stress in PVAT and limiting eNOS activity, making it a clear example of inside-to-outside signaling [85]. This condition eventually leads to the dysfunction of PVAT, in which its adipokine profile shifts toward hyperleptinemia, and increased TNF, IL-6, and resistin, all of which converge on impaired NO bioavailability and enhanced endothelin-1 and ROS-mediated endothelial dysfunction [81, 84, 86]. Chronic leptin excess, TNF/JNK–driven ET-1 production, IL-6–mediated eNOS inhibition, resistin-induced inflammatory cytokine release and insulin resistance collectively transform PVAT into a pro-inflammatory source that accelerates atherosclerosis and cardiovascular disease [87–89].

The endothelium in CAD patients also produces chemokines such as RANTES, CCL2 and CXCL10 and with the pro-inflammatory cytokines they orchestrate the recruitment of monocytes, T cells, B cells, dendritic and NK cells, thus facilitating inflammation in the artery wall [90, 91]. Emerging evidence suggests that PVAT may further amplify this chemokine-driven immune-cell recruitment. Chen et al. analyzed the GEO dataset GSE45169 and demonstrated increased expression of CD55⁺ADSC and CXCL14⁺ADSC marker genes in coronary PVAT compared with subcutaneous adipose tissue, indicating activation of these ADSC subtypes within the PVAT [40]. Consistently, subsequent single-cell analysis of human carotid PVAT by the same group identified CXCL14⁺ADSCs as a chemotactically active population that promotes CXCR4-dependent signaling in monocytes and other immune-cell subsets through the secretion of CXCL12 and CXCL14 [40]. The infiltration of monocytes into the adipose tissue is followed by their differentiation to adipose tissue macrophages and their polarization to pro-inflammatory M1-like macrophages. ScRNA-seq in rat PVAT first revealed that *Pdpn* + macrophages selectively express Phospholipase A2 Group IID (Pla2g2d), a secreted phospholipase implicated in lipid mediator generation. Functional in vitro assays showed that Pla2g2d acts on adipocyte-derived extracellular vesicles to liberate the ω-3 fatty acids DHA and EPA, a finding confirmed by targeted GC–MS lipidomics [34]. Inhibition of extracellular vesicle release or knockdown of Pla2g2d abolished DHA/EPA production, establishing macrophage Pla2g2d as the initiating component of the axis. Downstream effects were tested in primary PVAT adipocytes isolated from diabetic rats, where DHA/EPA treatment suppressed TNF, IL-6, MCP-1, resistin, and angiotensinogen/AngII expression, while increasing anti-inflammatory adipokines such as adiponectin, IL-10, and omentin. Mechanistically, ω-3 fatty acids activate GPR120 on adipocytes, triggering PI3K–Akt signaling and inhibiting NF-κB, and knockdown of GPR120 eliminated these metabolic and inflammatory responses [34].

PVAT communicates with the vascular wall not only through adipokines and lipid mediators but also through a diverse repertoire of non-coding RNAs that shape endothelial function, smooth muscle behaviour, and macrophage lipid handling.

Microarray-based profiling of human aortic PVAT identified a panel of long non-coding RNAs, including GAS5, H19, MIR22HG, IL6-AS1, LINC01180, ADAMTS9-AS1, DLGAP1-AS2, NUTM2B-AS1, FAM230A, and UBQLN1-AS1, whose expression strongly correlates with immune-cell infiltration and activation of TNF/NF-κB–dependent inflammatory pathways, hypoxia signaling via HIF-1, and PI3K–Akt activity [92]. Their regulatory potential is further supported by predicted or experimentally validated subcellular localization: MIR1-1HG, enriched in exosome-localized transcripts, appears poised for export into the vascular microenvironment, while GAS5, H19, and MIR22HG are hypoxia-responsive in endothelial cells, linking PVAT lncRNA signatures directly to endothelial stress responses and dysfunction.

This transcriptional landscape extends into the secretome, where PVAT-derived extracellular vesicles carry miRNAs with functional impact on vascular cells. One of the examples is miR-221-3p, a PVAT-derived miRNA shown to modulate vascular contractility in the setting of obesity-induced inflammation [93], illustrating how vesicles-borne RNAs can directly influence SMC tone. Another key mediator is miR-382-5p, whose levels are reduced in exosomes derived from PVAT of patients with coronary atherosclerosis [94]. Functional studies demonstrated that miR-382-5p enhances cholesterol efflux in macrophages by upregulating ABCA1 and ABCG1; its loss abolishes the anti-foam-cell effects normally conferred by PVAT exosomes. These actions require intact BMP4–PPARγ signaling, establishing a mechanistic axis: PVAT-EXO → miR-382-5p → BMP4/PPARγ → ABCA1/ABCG1, that is disrupted when PVAT becomes dysfunctional.

Another layer is provided by miR-206-3p, identified within stromal cell-derived PVAT extracellular vesicles and associated with pro-angiogenic and atheroprotective activity [95]. miR-206-3p enhances ABCA1-dependent cholesterol efflux, reduces macrophage lipid accumulation, and suppresses endothelial inflammatory activation. In mouse vascular injury models, this translates into improved wall stability and reduced macrophage infiltration, suggesting that miR-206-3p regulates a coordinated program of lipid handling and inflammation relevant to plaque progression [95].

Together, these findings reveal that PVAT ncRNAs have influence in vascular homeostasis and disease, showing promise for future research of biomarkers and therapeutic targets in cardiometabolic pathology.

### PVAT control of cell differentiation, survival, and matrix remodeling

Multi-omics analyses of PVAT demonstrate that this tissue actively shapes the cell cycle, differentiation programs, and survival of multiple vascular and stromal cell types, functioning as a dynamic regulatory hub rather than a passive fat depot. Single-cell transcriptomics of cPVAT of CAD patients, combined with ligand–receptor interaction mapping, reveals that *SPP1* + macrophages act as dominant signal senders within the PVAT microenvironment, releasing SPP1, IL1B, CCL2/8, and matrix-associated ligands such as COL1A1/2 and FN1, which strongly activate APOE^hi fibro-adipogenic progenitors (FAPs) toward extracellular-matrix remodeling and TGF-β–linked fibrotic pathways [35]. These macrophage-derived cues shift FAPs into a more proliferative and fibrotic state, while parallel signaling toward endothelial and smooth muscle cells promotes endothelial activation and smooth muscle phenotypic switching. Through these coordinated interactions, PVAT orchestrates inflammatory, proliferative, and structural remodeling processes that directly influence vascular homeostasis.

Insight into PVAT cell differentiation is further supported by metabolomics studies of PVAT-derived adipose stem cells (PV-ADSCs), which show that smooth muscle–like differentiation requires rapid and extensive metabolic rewiring. Within 24 h of TGF-β1 stimulation, NMR profiling detects broad shifts in intracellular metabolites in cell cultures, and subsequent GC–MS measurements confirm increased levels of TCA cycle intermediates, higher oxygen consumption, elevated phosphoenolpyruvate and lactate, and substantial enrichment in lipid metabolites including cholesterol [41]. These coordinated metabolic changes indicate that glycolysis, oxidative phosphorylation and lipid remodeling are activated at the onset of lineage commitment. The same metabolic program is induced by miR-378a-3p, a BAT enriched miRNA [96], and inhibited when this miRNA is suppressed [41] suggesting it to be a key upstream mediator of the PV-ADSC to SMC-like cell differentiation. These findings highlight that BAT-associated genetic programs within PVAT are also functionally significant in cell differentiation and supporting the vascular wall stability. In a single-cell atlas of human carotid PVAT, Chen et al. identified two functionally distinct ADSC subsets, CD55⁺ADSCs and CXCL14⁺ADSCs. Functional experiments suggested that these cells can migrate toward vascular lesions, contribute to endothelial-like lineages, and promote neointimal formation, pathological angiogenesis and smooth muscle cell phenotypic switching, at least partly through FGF2 secretion [40]. Phenotypic switching of VSMCs and aortic dilatation are also shown to be influenced by Liver kinase b1 (Lkb1) expression in animal PVAT, due to its regulation of renin-angiotensin system [97].

Proteomic and transcriptomic integration from healthy rodent tPVAT and aPVAT further highlights how PVAT regulates vascular smooth muscle cell survival, revealing that the secreted protein Cartilage oligomeric matrix protein (COMP) acts as a selective inhibitor of VSMCs apoptosis [43]. In functional assays, COMP reduces TNF/cycloheximide-induced apoptosis of VSMCs by decreasing cleaved PARP and cleaved caspase-3 while selectively increasing the anti-apoptotic protein survivin, without affecting Bcl-2. This survivin-dependent mechanism suggests that PVAT modulates the balance between VSMCs survival and death, a process central to plaque stability since excessive SMC apoptosis contributes to cap thinning and vulnerability. Together, these multi-omics findings position PVAT as an important orchestrator of vascular cell behaviour.

### PVAT-driven modulation of vascular tone and contractility

Omics analyses consistently show that PVAT is a key modulator of vascular contractility, integrating inflammatory signaling networks with lipid-derived paracrine factors that regulate SMC tone. Single-cell ligand–receptor profiling in animal models of type 2 diabetes demonstrates that in diabetes PVAT becomes a hyper-communicative signaling hub, with a global increase in the number and strength of intercellular interaction pairs [34]. Pathways such as VEGF, CXCL, and IL-1 become markedly upregulated, and macrophages engage in reciprocal activation loops with T cells, NK cells, fibroblasts, ECs and SMCs. These circuits amplify inflammation and promote structural remodeling of the vessel wall, with direct consequences for its contractile properties. A striking shift in cell-to-cell communication is observed: fibroblasts, which predominantly act as signal receivers in healthy PVAT, become major signal senders in diabetic PVAT, transmitting ANGPTL-, activin-, and TGF-β–related cues that alter endothelial function and bias SMCs toward a migratory, synthetic phenotype. Correspondingly, SMCs became major signal recipients, with upregulation of Tgfbr1/Tgfbr2 pathways linked to migration, contractile-synthetic switching, and insulin-related responses [34]. This concept is further supported by recent single-cell evidence from human carotid PVAT showing that CD55⁺ adipose-derived stem cells secrete FGF2 and promote VSMC phenotypic switching toward a synthetic state, linking PVAT progenitor-cell signaling to structural remodeling processes that may secondarily affect vascular contractile behavior [40]. Consistent with this line, PVAT-derived chemerin has also been shown to potentiate NE- and 5-HT–induced vasoconstriction and promote aortic stiffening in a sex-dependent manner, as chemerin1 receptor inhibition or genetic deletion attenuates these contractile responses, in male rats [98].

These transcriptomic insights align with lipidomic and functional studies showing that healthy PVAT releases a repertoire of lipid mediators that directly modulate SMC membrane potential and vascular tone. Lipidomic and bioassay profiling of animal PVAT revealed methyl palmitate (PAME), a product of differentiated PVAT adipocytes shown to induce vasodilation by hyperpolarizing SMCs through 4-AP–sensitive voltage-gated K channels [99]. However, in hypertension, PAME release is diminished, and PVAT instead promotes vasoconstriction through increased angiotensin II production. Recent lipidomic profiling expanded this framework by identifying oxylipins, such as 12,13-DiHOME, 9,10-DiHOME, and multiple HETEs, as perivascular relaxing factors (PVRFs) capable of enhancing vasodilation in resistance vessels [36]. Unlike PAME, these oxylipins act indirectly by facilitating opening of KCNQ (Kv7) family channels, particularly KCNQ5, thereby stabilizing membrane potential and promoting relaxation independently of NO/cGMP or cAMP signaling.

In addition to PVRFs, PVAT also produces PVAT-derived contracting factors (PVCFs), including superoxide anion, catecholamines, prostaglandins, angiotensin II, and resistin, that counteract PVRFs by reducing NO bioavailability and enhancing endothelial dysfunction [100–102]. The balance between PVRFs and PVCFs therefore determines whether PVAT exerts an anticontractile or pro-contractile influence on the adjacent vessel.

The importance of lipid homeostasis in shaping this balance was further explored using genetic models. In adipose triglyceride lipase (ATGL) deficient mice, PVAT loses its normal anti-contractile effect and no longer attenuates phenylephrine- or U46619-induced vasoconstriction, showing that adipocyte ATGL-dependent lipolysis is required for PVAT to modulate VSMCs contractile responses [103]. Adipocyte-specific restoration of ATGL fully rescues this phenotype, demonstrating that PVAT-vascular crosstalk relies on lipolysis-driven paracrine factors originating specifically from adipocytes within PVAT. Taken together, these omics-based findings reveal a unified model: PVAT regulates vascular contractility through an interplay of inflammatory signaling networks, lipidomic mediators, and adipocyte metabolic pathways, and disturbances in any of these layers, whether through diabetes-driven intercellular signaling shifts, altered lipid mediator synthesis, or impaired lipolysis, translate into pathological changes in vascular tone.

## Translational implications and challenges: therapeutic targeting of the PVAT–plaque crosstalk

Atherosclerotic cardiovascular disease (CVD) remains a major clinical challenge, particularly in patients with established disease, where current therapies insufficiently address the mechanisms driving plaque instability and subsequent MACEs such as myocardial infarction and stroke. This unmet need is further compounded by the rising global prevalence of obesity and T2DM, conditions that not only accelerate atherosclerosis but also introduce additional, incompletely understood risk modifiers. Identifying and mechanistically characterizing these emerging contributors is therefore critical for improving risk stratification and therapeutic targeting.

As discussed above, PVAT has emerged as a biologically active tissue compartment in close anatomical proximity to the vascular wall, with the capacity to influence plaque development and phenotype through paracrine and endocrine signaling. Importantly, clinical studies have demonstrated that PVAT undergoes disease-specific phenotypic remodeling in the setting of coronary artery disease, reflecting local vascular inflammation [104]. These changes can be captured non-invasively and have been shown to associate with plaque vulnerability and adverse cardiovascular outcomes.

Over the past decade, Antoniades and colleagues have pioneered imaging-based approaches to characterize PVAT using coronary computed tomography angiography (CCTA) [105]. Their development of the fat attenuation index (FAI) enables quantitative assessment of inflammation-induced alterations in PVAT composition, providing a surrogate readout of coronary vascular inflammation. Data from prospective cohorts, including the CRISP-CT (Cardiovascular RIsk Prediction using Computed Tomography) study, have established that elevated perivascular FAI independently stratifies risk for major adverse cardiovascular events, including cardiac mortality, beyond traditional risk factors, coronary artery calcium score, and high-risk plaque features. Notably, elevated FAI predicted events even among individuals with low or zero coronary calcium, highlighting its ability to detect biologically active disease in subclinical stages [106]. In addition to FAI, advanced radiotranscriptomic and machine learning-derived imaging signatures of PVAT have been developed that integrate radiomic texture, attenuation, and geometric features with underlying transcriptomic and histologic profiles. These multimodal biomarker, such as the fat radiomic profile (FRP), capture additional facets of PVAT remodeling, including fibrosis and microvascular alterations, which confer incremental prognostic value over FAI alone and improve prediction of adverse outcomes in patients with coronary artery disease [107]. These findings support a paradigm shift in risk stratification from anatomy-centric to biology-driven models and illustrate the potential clinical utility of PVAT phenotyping in primary and secondary prevention.

Despite remarkable progress in imaging-based assessment of PVAT, the development of PVAT-specific circulating biomarkers remains a major challenge. Although PVAT exhibits a rich and dynamic secretome, many of its secreted factors overlap with those derived from other adipose tissue depots. Consequently, adipokines such as adiponectin or leptin lack the spatial specificity required to reflect PVAT-localized inflammation surrounding diseased vessels [108]. In this context, multiomics profiling of PVAT holds significant promise. Deep transcriptomic, proteomic, lipidomic, and epigenomic analyses may enable the identification of PVAT-specific biosignatures that could be translated into multimarker panels for clinical liquid biopsy applications.

Beyond its value as a diagnostic and prognostic biomarker, accumulating evidence suggests that PVAT is a modifiable tissue compartment that responds dynamically to pharmacological intervention. Recent evidence revealed that several cardiometabolic drug classes, including DPP-4 inhibitors, GLP-1 receptor agonists, and SGLT-2 inhibitors, consistently attenuate PVAT inflammation and restore its vasorelaxant phenotype. These agents converge on overlapping molecular pathways. DPP-4 inhibitors suppress PVAT-derived pro-inflammatory cytokines and chemokines while enhancing GLP-1/GLP-1R signaling and activating the Akt–eNOS [109–111], AMPK, and autophagy-dependent vasodilatory pathways. GLP-1 receptor agonists increase adiponectin, promote M2 macrophage polarization, reduce adipocyte lipid accumulation, and signal through PI3K/Akt, MAPK, cAMP/PKA/CREB, and eNOS pathways to limit PVAT inflammation [112]. Similarly, SGLT-2 inhibitors reduce PVAT chemokine release, oxidative stress, and cytokine production, improving stromal cell function and vascular compliance [113]. These findings have fuelled interest in therapies that directly target PVAT, prompting development of adipose-specific delivery strategies, including targeting adipose-enriched genes such as ADIPOQ and PHB1 [114], adipocyte-binding aptamers [115, 116] and nanoparticle-based platforms with preferential adipose accumulation [117, 118]. Omics profiling of PVAT-derived EVs has further revealed enrichment of regulatory miRNAs and proteins that enhance cholesterol efflux, mitigate endothelial injury, and modulate immune cell function [93–95]. Building on these insights, neutrophil membrane–engineered EVs were developed to deliver PVAT-derived miRNA cargo, such as miR-206-3p, which promotes ABCA1-dependent cholesterol efflux, specifically to inflamed vascular sites, resulting in reduced vascular inflammation, plaque stabilization, and improved endothelial repair in preclinical models [95]. Finally, RNA-based approaches such as self-amplifying RNA (saRNA), which activate endogenous gene expression, offer a complementary strategy to selectively upregulate PVAT-relevant genes including ADIPOQ and PHB1, providing a novel therapeutic avenue for modulating PVAT function in cardiometabolic disease [114].

Realizing the full therapeutic and diagnostic potential of PVAT requires a comprehensive understanding of its cellular composition, spatial organization, and functional interactions with the vascular wall. Studying PVAT biology and its crosstalk with atherosclerotic plaques, however, remains technically and conceptually challenging. PVAT is a highly heterogeneous tissue composed of diverse adipocyte subtypes, immune cells, stromal cells, and vascular components, all of which may differentially contribute to plaque modulation. Comprehensive characterization of this complexity is hindered by limited tissue availability. In commonly used rodent models of atherosclerosis, coronary arteries and their surrounding PVAT are exceedingly small and largely inaccessible, necessitating reliance on aortic PVAT. While informative, these models have limited translational relevance due to fundamental developmental and functional differences between the aorta and coronary arteries. Conversely, access to human coronary PVAT is typically restricted to explanted or severely diseased hearts, while carotid endarterectomy specimens yield only minimal amounts of PVAT, often insufficient for extensive cellular or functional analyses.

To address these challenges, increasing attention has been directed toward multiomics approaches, which enable complementary characterization of the cellular, molecular, and metabolic landscape of PVAT. Nevertheless, despite this considerable potential a major limitation is that each omics layer reflects only a single dimension of PVAT biology. Additionally, each platform is influenced by distinct methodological conditions and technical constraints. Genomics can identify variants associated with specific metabolic traits, but does not directly define functional mechanisms. Transcriptomics, including single-cell approaches, resolves cell states and cellular heterogeneity, but may be influenced by sampling, tissue dissociation and disease-stage variability [119]. Proteomics provides closer insight into secreted, signaling and extracellular-matrix-associated mediators, although it is limited by protein abundance, post-translational modifications and spatial resolution [120]. Metabolomics and lipidomics offer functional information on PVAT metabolism and bioactive lipid signaling, but are particularly sensitive to diet, medication use, tissue handling and analytical conditions [121].

Most currently available omics datasets represent static molecular snapshots, which may be insufficient to capture the highly dynamic nature of PVAT biology during progressive atherosclerosis, plaque destabilization, and cardiovascular remodeling. Although integrated multiomics strategies may help link genetic, transcriptional, proteomic, and metabolic alterations into a more comprehensive understanding of PVAT function, their implementation remains computationally and biologically challenging. Differences in data dimensionality, normalization approaches, statistical modeling, and bioinformatic pipelines may substantially influence final results. Moreover, distinguishing causal molecular drivers from secondary epiphenomena remains difficult, particularly in cross-sectional human studies [122].

Current omics research is further limited by relatively small sample sizes, insufficient external validation, reliance on animal models, and underrepresentation of longitudinal clinical cohorts, restricting causal inference and clinical translation. Advancing PVAT omics research toward clinical application will require standardized tissue acquisition and analytical protocols, harmonized bioinformatic frameworks, integration with imaging-based assessments of PVAT inflammation and vascular remodeling, and validation in prospective multicenter cohorts linking omics-derived signatures with plaque vulnerability, cardiovascular outcomes, and therapeutic response [123]. Ultimately, translation into routine cardiovascular practice will also depend on reproducibility, cost-effectiveness, scalability, and the ability of omics-derived markers to provide incremental value beyond existing clinical and imaging risk stratification tools.

These limitations underscore the urgent need for innovative experimental models capable of recapitulating PVAT-plaque interactions in a controlled and physiologically relevant manner. In this regard, organ-on-chip and vascular microphysiological systems represent a particularly promising avenue. Recent advances have demonstrated the feasibility of modeling complex aspects of atherosclerosis, including endothelial dysfunction, immune cell recruitment, and shear stress–dependent signaling [124, 125]. To date, however, incorporation of PVAT into such platforms has been largely unexplored. Integrating PVAT components into next-generation organ-on-chip systems could provide unprecedented opportunities to dissect bidirectional PVAT–vascular crosstalk, identify novel therapeutic targets, and accelerate translational discovery.

## Data Availability

Data sharing is not applicable to this article as no datasets were generated or analysed during the current study.

## Abbreviations

PVAT: Perivascular adipose tissue
MACE: Major adverse cardiovascular events
WAT: White adipose tissue
BAT: Brown adipose tissue
VSMC: Vascular smooth muscle cells
EC: Endothelial cells
TNF: Tumor necrosis factor
MCP1: Monocyte chemoattractant protein 1
SVF: Stromal vascular fraction
APCs: Adipocyte progenitor cells
UCP1: Uncoupling protein 1
BMP8B: Bone morphogenetic protein 8b
FGF21: Fibroblast growth factor 21
KNG2: Kininogen 2
VV: Vasa vasorum
4-HNE: 4-Hydroxynonenal
eNOS: Endothelial nitric oxide synthase
BH4: Tetrahydrobiopterin
AMPK: AMP activated protein kinase
Pla2g2d: Phospholipase A2 group IID
COMP: Cartilage oligomeric matrix protein
PAME: Methyl palmitate
PVRFs: Perivascular relaxing factors
PVCFs: PVAT derived contracting factors
ATGL: Adipose triglyceride lipase
CCTA: Coronary computed tomography angiography
FAI: Fat attenuation index
saRNA: Self amplifying RNA

## References

[1] Stark BA, DeCleene NK, Desai EC, Hsu JM, Johnson CO, Lara-Castor L, et al. Global, regional, and national burden of cardiovascular diseases and risk factors in 204 countries and territories, 1990–2023. JACC. 2025;86(22):2167–243. 10.1016/j.jacc.2025.08.015. 40990886

[2] Kawai K, Kawakami R, Finn AV, Virmani R. Differences in stable and unstable atherosclerotic plaque. Arterioscler Thromb Vasc Biol. 2024;44(7):1474–84. 10.1161/ATVBAHA.124.319396. 38924440

[3] Libby P. The changing landscape of atherosclerosis. Nature. 2021;592(7855):524–33. 10.1038/s41586-021-03392-8. 33883728

[4] Sopić M, Vladimirov S, Munjas J, Mitić T, Hall IF, Jusic A, et al. Targeting noncoding RNAs to treat atherosclerosis. Br J Pharmacol. 2025;182(2):220–45. 10.1111/bph.16412. 38720437

[5] Grundy SM, Stone NJ, Bailey AL, Beam C, Birtcher KK, Blumenthal RS, et al. 2018 AHA/ACC/AACVPR/AAPA/ABC/ACPM/ADA/AGS/APhA/ASPC/NLA/PCNA guideline on the management of blood cholesterol: executive summary: a report of the american college of cardiology/american heart association task force on clinical practice guidelines. J Am Coll Cardiol. 2019;73(24):3168–209. 10.1016/j.jacc.2018.11.002. 30423391

[6] Rroji M, Spahia N, Figurek A, Spasovski G. Targeting diabetic atherosclerosis: the role of GLP-1 receptor agonists, SGLT2 inhibitors, and nonsteroidal mineralocorticoid receptor antagonists in vascular protection and disease modulation. Biomedicines. 2025;13(3):728. 10.3390/biomedicines13030728PubMedPMID:40149704;PubMedCentralPMCID:PMC11940462. PMC11940462 40149704

[7] Hassan M. CANTOS: A breakthrough that proves the inflammatory hypothesis of atherosclerosis. Glob Cardiol Sci Pract. 2018. 10.21542/gcsp.2018.2. PMC5857062 29644229

[8] Nidorf SM, Eikelboom JW, Budgeon CA, Thompson PL. Low-dose colchicine for secondary prevention of cardiovascular disease. J Am Coll Cardiol. 2013;61(4):404–10. 10.1016/j.jacc.2012.10.027. 23265346

[9] Bouabdallaoui N, Tardif JC, Waters DD, Pinto FJ, Maggioni AP, Diaz R, et al. Time-to-treatment initiation of colchicine and cardiovascular outcomes after myocardial infarction in the Colchicine Cardiovascular Outcomes Trial (COLCOT). Eur Heart J. 2020;41(42):4092–9. 10.1093/eurheartj/ehaa659. PMC7700755 32860034

[10] Zhang RS, Weber BN, Araiza-Garaygordobil D, Garshick MS. Colchicine for the prevention of cardiovascular disease: potential global implementation. Curr Cardiol Rep. 2024;26(5):423–34. 10.1007/s11886-024-02049-y. PMC11196186 38573553

[11] Bailey-Downs LC, Tucsek Z, Toth P, Sosnowska D, Gautam T, Sonntag WE, et al. Aging exacerbates obesity-induced oxidative stress and inflammation in perivascular adipose tissue in mice: a paracrine mechanism contributing to vascular redox dysregulation and inflammation. J Gerontol A Biol Sci Med Sci. 2013;68(7):780–92. 10.1093/gerona/gls238. PMC3674713 23213032

[12] Chatterjee TK, Stoll LL, Denning GM, Harrelson A, Blomkalns AL, Idelman G, et al. Proinflammatory phenotype of perivascular adipocytes: influence of high-fat feeding. Circ Res. 2009;104(4):541–9. 10.1161/CIRCRESAHA.108.182998. PMC2742882 19122178

[13] Nordestgaard LT, Wolford BN, de Gonzalo-Calvo D, Sopić M, Devaux Y, Matic L, et al. Multiomics in atherosclerotic cardiovascular disease. Atherosclerosis. 2025;408:120414. 10.1016/j.atherosclerosis.2025.120414. 40663987

[14] Antoniades C, Antonopoulos AS, Deanfield J. Imaging residual inflammatory cardiovascular risk. Eur Heart J. 2020;41(6):748–58. 10.1093/eurheartj/ehz474. 31317172

[15] Gil-Ortega M, Somoza B, Huang Y, Gollasch M, Fernández-Alfonso MS. Regional differences in perivascular adipose tissue impacting vascular homeostasis. Trends Endocrinol Metab. 2015;26(7):367–75. 10.1016/j.tem.2015.04.003. 26008879

[16] Tran KV, Fitzgibbons T, Min SY, DeSouza T, Corvera S. Distinct adipocyte progenitor cells are associated with regional phenotypes of perivascular aortic fat in mice. Mol Metab. 2018;9:199–206. 10.1016/j.molmet.2017.12.014. PMC5869733 29396370

[17] Gaborit B, Venteclef N, Ancel P, Pelloux V, Gariboldi V, Leprince P, et al. Human epicardial adipose tissue has a specific transcriptomic signature depending on its anatomical peri-atrial, peri-ventricular, or peri-coronary location. Cardiovasc Res. 2015;108(1):62–73. 10.1093/cvr/cvv208. 26239655

[18] Da Silva RDNO, Santos-Eichler RA, Dias C, Rodrigues SF, Skiba DS, Landgraf RG, et al. Immune spleen cells attenuate the inflammatory profile of the mesenteric perivascular adipose tissue in obese mice. Sci Rep. 2021;11(1):11153. 10.1038/s41598-021-90600-0. PMC8160359 34045574

[19] Li Y, Chen Z, Xiao Y, Li X. Cross-talks between perivascular adipose tissue and neighbors: multifaceted nature of nereids. Front Pharmacol. 2024;2(15):1442086. 10.3389/fphar.2024.1442086. PMC11327032 39156105

[20] Ozen G, Daci A, Norel X, Topal G. Human perivascular adipose tissue dysfunction as a cause of vascular disease: Focus on vascular tone and wall remodeling. Eur J Pharmacol. 2015;766:16–24. 10.1016/j.ejphar.2015.09.012. 26424111

[21] Antonopoulos AS, Sanna F, Sabharwal N, Thomas S, Oikonomou EK, Herdman L, et al. Detecting human coronary inflammation by imaging perivascular fat. Sci Transl Med. 2017;9(398):eaal2658. 10.1126/scitranslmed.aal2658. 28701474

[22] Takaoka M, Suzuki H, Shioda S, Sekikawa K, Saito Y, Nagai R, et al. Endovascular injury induces rapid phenotypic changes in perivascular adipose tissue. Arterioscler Thromb Vasc Biol. 2010;30(8):1576–82. 10.1161/ATVBAHA.110.207175. 20489168

[23] Ohyama K, Matsumoto Y, Amamizu H, Uzuka H, Nishimiya K, Morosawa S, et al. Association of coronary perivascular adipose tissue inflammation and drug-eluting stent-induced coronary hyperconstricting responses in pigs: 18F-fluorodeoxyglucose positron emission tomography imaging study. Arterioscler Thromb Vasc Biol. 2017;37(9):1757–64. 10.1161/ATVBAHA.117.309843. 28751570

[24] Qi XY, Qu SL, Xiong WH, Rom O, Chang L, Jiang ZS. Perivascular adipose tissue (PVAT) in atherosclerosis: a double-edged sword. Cardiovasc Diabetol. 2018;17(1):134. 10.1186/s12933-018-0777-x. PMC6180425 30305178

[25] Chang L, Villacorta L, Li R, Hamblin M, Xu W, Dou C, et al. Loss of perivascular adipose tissue upon PPARγ deletion in smooth muscle cells impairs intravascular thermoregulation and enhances atherosclerosis. Circulation. 2012;126(9):1067–78. 10.1161/CIRCULATIONAHA.112.104489PubMedPMID:22855570;PubMedCentralPMCID:PMC3493564. PMC3493564 22855570

[26] Ketonen J, Shi J, Martonen E, Mervaala E. Periadventitial adipose tissue promotes endothelial dysfunction via oxidative stress in diet-induced obese C57Bl/6 mice. Circ J Off J Jpn Circ Soc. 2010;74(7):1479–87. 10.1253/circj.cj-09-0661. 20526041

[27] Marchesi C, Ebrahimian T, Angulo O, Paradis P, Schiffrin EL. Endothelial nitric oxide synthase uncoupling and perivascular adipose oxidative stress and inflammation contribute to vascular dysfunction in a rodent model of metabolic syndrome. Hypertension. 2009;54(6):1384–92. 10.1161/HYPERTENSIONAHA.109.138305. 19822799

[28] Park SY, Kim KH, Seo KW, Bae JU, Kim YH, Lee SJ, et al. Resistin derived from diabetic perivascular adipose tissue up-regulates vascular expression of osteopontin via the AP-1 signalling pathway. J Pathol. 2014;232(1):87–97. 10.1002/path.4286. PMC4285806 24089355

[29] Chen Y, Wang X, Mai J, Zhao X, Liang Y, Gu M, et al. C-reactive protein promotes vascular endothelial dysfunction partly via activating adipose tissue inflammation in hyperlipidemic rabbits. Int J Cardiol. 2013;168(3):2397–403. 10.1016/j.ijcard.2013.01.158. 23452887

[30] Manka D, Chatterjee TK, Stoll LL, Basford JE, Konaniah ES, Srinivasan R, et al. Transplanted perivascular adipose tissue accelerates injury-induced neointimal hyperplasia: role of monocyte chemoattractant protein-1. Arterioscler Thromb Vasc Biol. 2014;34(8):1723–30. 10.1161/ATVBAHA.114.303983. PMC4103955 24947528

[31] Schroeter MR, Eschholz N, Herzberg S, Jerchel I, Leifheit-Nestler M, Czepluch FS, et al. Leptin-dependent and leptin-independent paracrine effects of perivascular adipose tissue on neointima formation. Arterioscler Thromb Vasc Biol. 2013;33(5):980–7. 10.1161/ATVBAHA.113.301393. 23520165

[32] Li H, Wang YP, Zhang LN, Tian G. Perivascular adipose tissue-derived leptin promotes vascular smooth muscle cell phenotypic switching via p38 mitogen-activated protein kinase in metabolic syndrome rats. Exp Biol Med. 2014;239(8):954–65. 10.1177/1535370214527903. 24719379

[33] Verhagen SN, Vink A, Van Der Graaf Y, Visseren FLJ. Coronary perivascular adipose tissue characteristics are related to atherosclerotic plaque size and composition. A post-mortem study Atherosclerosis. 2012;225(1):99–104. 10.1016/j.atherosclerosis.2012.08.031. 23022141

[34] Li J, Tian Z, Zhang T, Jin J, Zhang X, Xie P, et al. Single-cell view and a novel protective macrophage subset in perivascular adipose tissue in T2DM. Cell Mol Biol Lett. 2024;29(1):148. 10.1186/s11658-024-00668-5. PMC11616190 39627688

[35] Fu M, Shu S, Peng Z, Liu X, Chen X, Zeng Z, et al. Single-cell RNA sequencing of coronary perivascular adipose tissue from end-stage heart failure patients identifies *SPP1*^+^ macrophage subpopulation as a target for alleviating fibrosis. Arterioscler Thromb Vasc Biol. 2023;43(11):2143–64. 10.1161/ATVBAHA.123.319828. PMC10597444 37706320

[36] Tsvetkov D, Schleifenbaum J, Wang Y, Kassmann M, Polovitskaya MM, Ali M, et al. KCNQ5 controls perivascular adipose tissue-mediated vasodilation. Hypertension. 2024;81(3):561–71. 10.1161/HYPERTENSIONAHA.123.21834. 38354270

[37] Arderiu G, Bejar MT, Civit-Urgell A, Peña E, Badimon L. Crosstalk of human coronary perivascular adipose-derived stem cells with vascular cells: role of tissue factor. Basic Res Cardiol. 2024;119(2):291–307. 10.1007/s00395-024-01037-1. 38430261

[38] Rendon CJ, Sempere L, Lauver A, Watts SW, Contreras GA. Anatomical location, sex, and age modulate adipocyte progenitor populations in perivascular adipose tissues. Front Physiol. 2024;12(15):1411218. 10.3389/fphys.2024.1411218. PMC11282503 39072214

[39] Angueira AR, Sakers AP, Holman CD, Cheng L, Arbocco MN, Shamsi F, et al. Defining the lineage of thermogenic perivascular adipose tissue. Nat Metab. 2021;3(4):469–84. 10.1038/s42255-021-00380-0. PMC8136151 33846639

[40] Chen J, Li K, Shao J, Mei S, Lai Z, Zhao H, et al. Perivascular adipose single-cell atlas identifies CD55+ adipose-derived stem cells as vascular remodeling regulators in atherosclerosis. Nat Commun. 2026. 10.1038/s41467-026-72962-z. PMC13376731 42135279

[41] Gu W, Nowak WN, Xie Y, Le Bras A, Hu Y, Deng J, et al. Single-Cell RNA-sequencing and metabolomics analyses reveal the contribution of perivascular adipose tissue stem cells to vascular remodeling. Arterioscler Thromb Vasc Biol. 2019;39(10):2049–66. 10.1161/ATVBAHA.119.312732. PMC6766361 31340667

[42] Soucy A, Potts C, Kaija A, Harrington A, McGilvrey M, Sutphin GL, et al. Effects of a Global *Rab27a* null mutation on murine PVAT and cardiovascular function. Arterioscler Thromb Vasc Biol. 2024;44(7):1601–16. 10.1161/ATVBAHA.124.320969. PMC11209784 38660803

[43] Huang C, Huang Y, Yao L, Li J, Zhang Z, Huang Z, et al. Thoracic perivascular adipose tissue inhibits VSMC apoptosis and aortic aneurysm formation in mice via the secretome of browning adipocytes. Acta Pharmacol Sin. 2023;44(2):345–55. 10.1038/s41401-022-00959-7. PMC9889802 35945313

[44] Trapnell C, Cacchiarelli D, Grimsby J, Pokharel P, Li S, Morse M, et al. The dynamics and regulators of cell fate decisions are revealed by pseudotemporal ordering of single cells. Nat Biotechnol. 2014;32(4):381–6. 10.1038/nbt.2859. PMC4122333 24658644

[45] Iacobellis G. Local and systemic effects of the multifaceted epicardial adipose tissue depot. Nat Rev Endocrinol. 2015;11(6):363–71. 10.1038/nrendo.2015.58. 25850659

[46] Kumar RK, Yang Y, Contreras AG, Garver H, Bhattacharya S, Fink GD, et al. Phenotypic changes in T cell and macrophage subtypes in perivascular adipose tissues precede high-fat diet-induced hypertension. Front Physiol. 2021;17(12):616055. 10.3389/fphys.2021.616055. PMC8010306 33815135

[47] Skiba DS, Nosalski R, Mikolajczyk TP, Siedlinski M, Rios FJ, Montezano AC, et al. Anti-atherosclerotic effect of the angiotensin 1–7 mimetic AVE0991 is mediated by inhibition of perivascular and plaque inflammation in early atherosclerosis. Br J Pharmacol. 2017;174(22):4055–69. 10.1111/bph.13685. PMC5659999 27935022

[48] Ying Z, Tramper N, Zhou E, Boon MR, Rensen PCN, Kooijman S. Role of thermogenic adipose tissue in lipid metabolism and atherosclerotic cardiovascular disease: lessons from studies in mice and humans. Cardiovasc Res. 2023;119(4):905–18. 10.1093/cvr/cvac131. PMC10153643 35944189

[49] Padilla J, Jenkins NT, Vieira-Potter VJ, Laughlin MH. Divergent phenotype of rat thoracic and abdominal perivascular adipose tissues. Am J Physiol-Regul Integr Comp Physiol. 2013;304(7):R543–52. 10.1152/ajpregu.00567.2012. PMC3627942 23389108

[50] Aldiss P, Davies G, Woods R, Budge H, Sacks HS, Symonds ME. ‘Browning’ the cardiac and peri-vascular adipose tissues to modulate cardiovascular risk. Int J Cardiol. 2017;228:265–74. 10.1016/j.ijcard.2016.11.074. PMC5236060 27865196

[51] Chang L, Garcia-Barrio MT, Chen YE. Perivascular adipose tissue regulates vascular function by targeting vascular smooth muscle cells. Arterioscler Thromb Vasc Biol. 2020;40(5):1094–109. 10.1161/ATVBAHA.120.312464. PMC7441816 32188271

[52] Kim S, Lee ES, Lee SW, Kim YH, Lee CH, Jo DG, et al. Site-specific impairment of perivascular adipose tissue on advanced atherosclerotic plaques using multimodal nonlinear optical imaging. Proc Natl Acad Sci. 2019;116(36):17765–74. 10.1073/pnas.1902007116. PMC6731664 31427531

[53] Garcia-Carrizo F, Priego T, Szostaczuk N, Palou A, Picó C. Sexual dimorphism in the age-induced insulin resistance, liver steatosis, and adipose tissue function in rats. Front Physiol. 2017;11(8):445. 10.3389/fphys.2017.00445. PMC5504177 28744221

[54] Kong LR, Zhou YP, Chen DR, Ruan CC, Gao PJ. Decrease of perivascular adipose tissue browning is associated with vascular dysfunction in spontaneous hypertensive rats during aging. Front Physiol. 2018;18(9):400. 10.3389/fphys.2018.00400. PMC5915562 29720945

[55] Bussey CE, Withers SB, Saxton SN, Bodagh N, Aldous RG, Heagerty AM. β_3_ -Adrenoceptor stimulation of perivascular adipocytes leads to increased fat cell-derived NO and vascular relaxation in small arteries. Br J Pharmacol. 2018;175(18):3685–98. 10.1111/bph.14433. PMC6109217 29980164

[56] Mestres-Arenas A, Villarroya J, Giralt M, Villarroya F, Peyrou M. A differential pattern of batokine expression in perivascular adipose tissue depots from mice. Front Physiol. 2021;4(12):714530. 10.3389/fphys.2021.714530. PMC8373243 34421656

[57] Adachi Y, Ueda K, Nomura S, Ito K, Katoh M, Katagiri M, et al. Beiging of perivascular adipose tissue regulates its inflammation and vascular remodeling. Nat Commun. 2022;13(1):5117. 10.1038/s41467-022-32658-6. PMC9452496 36071032

[58] Schumacher MA, Hedl M, Abraham C, Bernard JK, Lozano PR, Hsieh JJ, et al. ErbB4 signaling stimulates pro-inflammatory macrophage apoptosis and limits colonic inflammation. Cell Death Dis. 2017;8(2):e2622–e2622. 10.1038/cddis.2017.42. PMC5386486 28230865

[59] Sacks HS, Fain JN. Human epicardial adipose tissue: a review. Am Heart J. 2007;153(6):907–17. 10.1016/j.ahj.2007.03.019. 17540190

[60] Fan W, Si Y, Xing E, Feng Z, Ding Z, Liu Y, et al. Human epicardial adipose tissue inflammation correlates with coronary artery disease. Cytokine. 2023;162:156119. 10.1016/j.cyto.2022.156119. 36603481

[61] Mazurek T, Zhang L, Zalewski A, Mannion JD, Diehl JT, Arafat H, et al. Human epicardial adipose tissue is a source of inflammatory mediators. Circulation. 2003;108(20):2460–6. 10.1161/01.CIR.0000099542.57313.C5. 14581396

[62] Taguchi R. Pericardial fat accumulation in men as a risk factor for coronary artery disease. Atherosclerosis. 2001;157(1):203–9. 10.1016/S0021-9150(00)00709-7. 11427222

[63] Ito H, Wakatsuki T, Yamaguchi K, Fukuda D, Kawabata Y, Matsuura T, et al. Atherosclerotic coronary plaque is associated with adventitial vasa vasorum and local inflammation in adjacent epicardial adipose tissue in fresh cadavers. Circ J. 2020;84(5):769–75. 10.1253/circj.CJ-19-0914. 32281556

[64] Virmani R, Kolodgie FD, Burke AP, Finn AV, Gold HK, Tulenko TN, et al. Atherosclerotic plaque progression and vulnerability to rupture. Arterioscler Thromb Vasc Biol. 2005;25(10):2054–61. 10.1161/01.ATV.0000178991.71605.18. 16037567

[65] Uchida Y, Uchida Y, Shimoyama E, Hiruta N, Kishimoto T, Watanabe S. Pericoronary adipose tissue as storage and supply site for oxidized low-density lipoprotein in human coronary plaques. PLoS ONE. 2016;11(3):e0150862. 10.1371/journal.pone.0150862. PMC4807097 27010927

[66] Michel JB, Virmani R, Arbustini E, Pasterkamp G. Intraplaque haemorrhages as the trigger of plaque vulnerability. Eur Heart J. 2011;32(16):1977–85. 10.1093/eurheartj/ehr054. PMC3155759 21398643

[67] Moreno PR, Purushothaman KR, Fuster V, Echeverri D, Truszczynska H, Sharma SK, et al. Plaque neovascularization is increased in ruptured atherosclerotic lesions of human aorta: implications for plaque vulnerability. Circulation. 2004;110(14):2032–8. 10.1161/01.CIR.0000143233.87854.23. 15451780

[68] Le Lay S, Scherer PE. Exploring adipose tissue-derived extracellular vesicles in inter-organ crosstalk: Implications for metabolic regulation and adipose tissue function. Cell Rep. 2025;44(6):115732. 10.1016/j.celrep.2025.115732. 40408250

[69] Zhou W, Zheng X, Cheng C, Guo G, Zhong Y, Liu W, et al. Rab27a deletion impairs the therapeutic potential of endothelial progenitor cells for myocardial infarction. Mol Cell Biochem. 2021;476(2):797–807. 10.1007/s11010-020-03945-x. 33095380

[70] Boucher JM, Robich M, Scott SS, Yang X, Ryzhova L, Turner JE, et al. Rab27a regulates human perivascular adipose progenitor cell differentiation. Cardiovasc Drugs Ther. 2018;32(5):519–30. 10.1007/s10557-018-6813-y. PMC6542275 30105417

[71] Chan CC, Damen MSMA, Alarcon PC, Sanchez-Gurmaches J, Divanovic S. Inflammation and immunity: from an adipocyte’s perspective. J Interferon Cytokine Res. 2019;39(8):459–71. 10.1089/jir.2019.0014. PMC6660836 30920343

[72] Kumada M, Kihara S, Ouchi N, Kobayashi H, Okamoto Y, Ohashi K, et al. Adiponectin specifically increased tissue inhibitor of metalloproteinase-1 through interleukin-10 expression in human macrophages. Circulation. 2004;109(17):2046–9. 10.1161/01.CIR.0000127953.98131.ED. 15096450

[73] Antonopoulos AS, Margaritis M, Coutinho P, Shirodaria C, Psarros C, Herdman L, et al. Adiponectin as a link between type 2 diabetes and vascular NADPH oxidase activity in the human arterial wall: the regulatory role of perivascular adipose tissue. Diabetes. 2015;64(6):2207–19. 10.2337/db14-1011. 25552596

[74] Margaritis M, Antonopoulos AS, Digby J, Lee R, Reilly S, Coutinho P, et al. Interactions between vascular wall and perivascular adipose tissue reveal novel roles for adiponectin in the regulation of endothelial nitric oxide synthase function in human vessels. Circulation. 2013;127(22):2209–21. 10.1161/CIRCULATIONAHA.112.001133. 23625959

[75] Fesus G, Dubrovska G, Gorzelniak K, Kluge R, Huang Y, Luft F, et al. Adiponectin is a novel humoral vasodilator. Cardiovasc Res. 2007;75(4):719–27. 10.1016/j.cardiores.2007.05.025. 17617391

[76] Almabrouk TAM, Ewart MA, Salt IP, Kennedy S. Perivascular fat, AMP‐activated protein kinase and vascular diseases. Br J Pharmacol. 2014;171(3):595–617. 10.1111/bph.12479. PMC3969075 24490856

[77] Man AWC, Zhou Y, Xia N, Li H. Endothelial nitric oxide synthase in the perivascular adipose tissue. Biomedicines. 2022;10(7):1754. 10.3390/biomedicines10071754. PMC9313312 35885059

[78] Zou L, Wang W, Liu S, Zhao X, Lyv Y, Du C, et al. Spontaneous hypertension occurs with adipose tissue dysfunction in perilipin-1 null mice. Biochim Biophys Acta. 2016;1862(2):182–91. 10.1016/j.bbadis.2015.10.024. 26521150

[79] Chen JY, Wu YP, Li CY, Jheng HF, Kao LZ, Yang CC, et al. PPARγ activation improves the microenvironment of perivascular adipose tissue and attenuates aortic stiffening in obesity. J Biomed Sci. 2021;28(1):22. 10.1186/s12929-021-00720-y. PMC8008548 33781257

[80] Kourliouros A, Karastergiou K, Nowell J, Gukop P, Tavakkoli Hosseini M, Valencia O, et al. Protective effect of epicardial adiponectin on atrial fibrillation following cardiac surgery. Eur J Cardiothorac Surg. 2011;39(2):228–32. 10.1016/j.ejcts.2010.05.006. 20609593

[81] Virdis A, Duranti E, Rossi C, Dell’Agnello U, Santini E, Anselmino M, et al. Tumour necrosis factor-alpha participates on the endothelin-1/nitric oxide imbalance in small arteries from obese patients: role of perivascular adipose tissue. Eur Heart J. 2015;36(13):784–94. 10.1093/eurheartj/ehu072. 24578389

[82] Didion S. Cellular and oxidative mechanisms associated with interleukin-6 signaling in the vasculature. Int J Mol Sci. 2017;18(12):2563. 10.3390/ijms18122563. PMC5751166 29186034

[83] Xia N, Li H. The role of perivascular adipose tissue in obesity-induced vascular dysfunction. Br J Pharmacol. 2017;174(20):3425–42. 10.1111/bph.13650. PMC5610151 27761903

[84] Xia N, Horke S, Habermeier A, Closs EI, Reifenberg G, Gericke A, et al. Uncoupling of endothelial nitric oxide synthase in perivascular adipose tissue of diet-induced obese mice. Arterioscler Thromb Vasc Biol. 2016;36(1):78–85. 10.1161/ATVBAHA.115.306263. 26586660

[85] Engin A. Endothelial Dysfunction in Obesity. In: Engin AB, Engin A, editors. Obesity and Lipotoxicity [Internet]. Cham: Springer International Publishing; 2017 [cited 2026 Jan 6]. p. 345–79. (Advances in Experimental Medicine and Biology). Available from: http://link.springer.com/10.1007/978-3-319-48382-5_15 28585207

[86] Gil-Ortega M, Condezo-Hoyos L, García-Prieto CF, Arribas SM, González MC, Aranguez I, et al. Imbalance between pro and anti-oxidant mechanisms in perivascular adipose tissue aggravates long-term high-fat diet-derived endothelial dysfunction. PLoS ONE. 2014;9(4):e95312. 10.1371/journal.pone.0095312. PMC3997398 24760053

[87] Raman P, Khanal S. Leptin in atherosclerosis: focus on macrophages, endothelial and smooth muscle cells. Int J Mol Sci. 2021;22(11):5446. 10.3390/ijms22115446. PMC8196747 34064112

[88] Jiang S, Park DW, Tadie JM, Gregoire M, Deshane J, Pittet JF, et al. Human resistin promotes neutrophil proinflammatory activation and neutrophil extracellular trap formation and increases severity of acute lung injury. J Immunol. 2014;192(10):4795–803. 10.4049/jimmunol.1302764. PMC4018664 24719460

[89] Reilly MP, Lehrke M, Wolfe ML, Rohatgi A, Lazar MA, Rader DJ. Resistin Is an inflammatory marker of atherosclerosis in humans. Circulation. 2005;111(7):932–9. 10.1161/01.CIR.0000155620.10387.43. 15710760

[90] Mateo T, Nabah Y, Abu-Taha M, Mata M, Cerdá-Nicolás M, Proudfoot A, et al. Angiotensin II-induced mononuclear leukocyte interactions with arteriolar and venular endothelium are mediated by the release of different CC chemokines. J Immunol. 2006;176(9):5577–86. 10.4049/jimmunol.176.9.5577. 16622027

[91] Ide N, Hirase T, Nishimoto-Hazuku A, Ikeda Y, Node K. Angiotensin II increases expression of IP-10 and the renin-angiotensin system in endothelial cells. Hypertens Res Off J Jpn Soc Hypertens. 2008;31(6):1257–67. 10.1291/hypres.31.1257. 18716376

[92] Xie X, Wang S, Rao J, Xue J, Lin K, Lin N, et al. Comprehensive analysis of differentially expressed lncRNAs in the perivascular adipose tissue of patients with coronary heart disease. Rev Cardiovasc Med. 2022;23(10):341. 10.31083/j.rcm2310341. PMC11267359 39077137

[93] Li X, Ballantyne LL, Yu Y, Funk CD. Perivascular adipose tissue–derived extracellular vesicle miR-221-3p mediates vascular remodeling. FASEB J. 2019;33(11):12704–22. 10.1096/fj.201901548R. PMC6902668 31469602

[94] Liu Y, Sun Y, Lin X, Zhang D, Hu C, Liu J, et al. Perivascular adipose-derived exosomes reduce macrophage foam cell formation through miR-382-5p and the BMP4-PPARγ-ABCA1/ABCG1 pathways. Vascul Pharmacol. 2022;143:106968. 10.1016/j.vph.2022.106968. 35123060

[95] Wei X, Guan X, Ma X, He X, Wang S, Zhou G, et al. Neutrophil membrane-engineered extracellular vesicles from perivascular adipose tissue-derived stromal cells for atherosclerotic lesion resolution and enhanced vascular graft remodeling. Adv Funct Mater. 2025;35(47):2503873. 10.1002/adfm.202503873.

[96] Xu J, Cui L, Wang J, Zheng S, Zhang H, Ke S, et al. Cold-activated brown fat-derived extracellular vesicle-miR-378a-3p stimulates hepatic gluconeogenesis in male mice. Nat Commun. 2023;14(1):5480. 10.1038/s41467-023-41160-6. PMC10482845 37673898

[97] Cai Z, Liang M, Jiang Y, Chen H, Hu Y, Huang Y, et al. Lkb1 downregulation links PVAT remodeling to aortic dilation or aneurysm. Circ Res. 2025;137(7):1006–23. 10.1161/CIRCRESAHA.125.326297. 40874959

[98] Wabel EA, Krieger-Burke T, Watts SW. Vascular chemerin from PVAT contributes to norepinephrine and serotonin-induced vasoconstriction and vascular stiffness in a sex-dependent manner. Am J Physiol Heart Circ Physiol. 2024;327(6):H1577–89. 10.1152/ajpheart.00475.2024. 39453435

[99] Lee YC, Chang HH, Chiang CL, Liu CH, Yeh JI, Chen MF, et al. Role of perivascular adipose tissue–derived methyl palmitate in vascular tone regulation and pathogenesis of hypertension. Circulation. 2011;124(10):1160–71. 10.1161/CIRCULATIONAHA.111.027375. 21844078

[100] Gao YJ, Takemori K, Su LY, An WS, Lu C, Sharma AM, et al. Perivascular adipose tissue promotes vasoconstriction: the role of superoxide anion. Cardiovasc Res. 2006;71(2):363–73. 10.1016/j.cardiores.2006.03.013. 16756966

[101] Ayala-Lopez N, Martini M, Jackson WF, Darios E, Burnett R, Seitz B, et al. Perivascular adipose tissue contains functional catecholamines. Pharmacol Res Perspect. 2014;2(3):e00041. 10.1002/prp2.41. PMC4041285 24904751

[102] Lee MHH, Chen SJ, Tsao CM, Wu CC. Perivascular adipose tissue inhibits endothelial function of rat aortas via caveolin-1. PLoS ONE. 2014;9(6):e99947. 10.1371/journal.pone.0099947. PMC4057398 24926683

[103] Schrammel A, Wölkart G, Ableitner E, Derler M, Potoschnig I, Schoiswohl G, et al. Adipose triglyceride lipase knockout increases anticontractile effects of perivascular adipose tissue. Arterioscler Thromb Vasc Biol. 2025;45(12):2196–209. 10.1161/ATVBAHA.125.322902. PMC12643563 41164878

[104] Antonopoulos AS, Antoniades C. Perivascular fat attenuation index by computed tomography as a metric of coronary inflammation. J Am Coll Cardiol. 2018;71(23):2708–9. 10.1016/j.jacc.2018.03.511. 29880135

[105] Kotanidis CP, Antoniades C. Perivascular fat imaging by computed tomography (CT): a virtual guide. Br J Pharmacol. 2021;178(21):4270–90. 10.1111/bph.15634PubMedPMID:34296764;PubMedCentralPMCID:PMC8856184. PMC8856184 34296764

[106] Oikonomou EK, Desai MY, Marwan M, Kotanidis CP, Antonopoulos AS, Schottlander D, et al. Perivascular fat attenuation index stratifies cardiac risk associated with high-risk plaques in the CRISP-CT study. J Am Coll Cardiol. 2020;76(6):755–7. 10.1016/j.jacc.2020.05.078. 32762910

[107] Oikonomou EK, Williams MC, Kotanidis CP, Desai MY, Marwan M, Antonopoulos AS, et al. A novel machine learning-derived radiotranscriptomic signature of perivascular fat improves cardiac risk prediction using coronary CT angiography. Eur Heart J. 2019;40(43):3529–43. 10.1093/eurheartj/ehz592. PMC6855141 31504423

[108] Antoniades C, Tousoulis D, Vavlukis M, Fleming I, Duncker DJ, Eringa E, et al. Perivascular adipose tissue as a source of therapeutic targets and clinical biomarkers: a clinical consensus statement from the European Society of Cardiology Working Group on Coronary Pathophysiology and Micro-circulation. Eur Heart J. 2023;44(38):3827–44. 10.1093/eurheartj/ehad484. PMC10568001 37599464

[109] Akoumianakis I, Antoniades C. Dipeptidyl peptidase IV inhibitors as novel regulators of vascular disease. Vascul Pharmacol. 2017;96–98:1–4. 10.1016/j.vph.2017.07.001. 28697993

[110] Zhu B, Li Y, Mei W, He M, Ding Y, Meng B, et al. Alogliptin improves endothelial function by promoting autophagy in perivascular adipose tissue of obese mice through a GLP-1-dependent mechanism. Vascul Pharmacol. 2019;115:55–63. 10.1016/j.vph.2018.11.003. 30447331

[111] Salim HM, Fukuda D, Higashikuni Y, Tanaka K, Hirata Y, Yagi S, et al. Teneligliptin, a dipeptidyl peptidase-4 inhibitor, attenuated pro-inflammatory phenotype of perivascular adipose tissue and inhibited atherogenesis in normoglycemic apolipoprotein-E-deficient mice. Vascul Pharmacol. 2017;96–98:19–25. 10.1016/j.vph.2017.03.003. 28347868

[112] Han F, Hou N, Liu Y, Huang N, Pan R, Zhang X, et al. Liraglutide improves vascular dysfunction by regulating a cAMP-independent PKA-AMPK pathway in perivascular adipose tissue in obese mice. Biomed Pharmacother. 2019;120:109537. 10.1016/j.biopha.2019.109537. 31605951

[113] Mori Y, Terasaki M, Hiromura M, Saito T, Kushima H, Koshibu M, et al. Luseogliflozin attenuates neointimal hyperplasia after wire injury in high-fat diet-fed mice via inhibition of perivascular adipose tissue remodeling. Cardiovasc Diabetol. 2019;18(1):143. 10.1186/s12933-019-0947-5. PMC6823953 31672147

[114] Liu D, Chen W, Guo Z, Gao Q, Wang B, Wang J, et al. Advances and challenges of targeting epicardial adipose tissue (EAT) and perivascular adipose tissue (PVAT). Cardiovasc Diabetol. 2025;24(1):319. 10.1186/s12933-025-02763-z. PMC12323123 40760703

[115] Liu J, Liu H, Sefah K, Liu B, Pu Y, Van Simaeys D, et al. Selection of aptamers specific for adipose tissue. PLoS ONE. 2012;7(5):e37789. 10.1371/journal.pone.0037789. PMC3360593 22662223

[116] Sakurai Y, Kajimoto K, Hatakeyama H, Harashima H. Advances in an active and passive targeting to tumor and adipose tissues. Expert Opin Drug Deliv. 2015;12(1):41–52. 10.1517/17425247.2015.955847. 25376864

[117] Hossen MdN, Kajimoto K, Akita H, Hyodo M, Ishitsuka T, Harashima H. Ligand-based targeted delivery of a peptide modified nanocarrier to endothelial cells in adipose tissue. J Controlled Release. 2010;147(2):261–8. 10.1016/j.jconrel.2010.07.100. 20647023

[118] Hsu CY, Liao CC, Lin ZC, Alalaiwe A, Hwang E, Lin TW, et al. Facile adipocyte uptake and liver/adipose tissue delivery of conjugated linoleic acid-loaded tocol nanocarriers for a synergistic anti-adipogenesis effect. J Nanobiotechnology. 2024;22(1):50. 10.1186/s12951-024-02316-8. PMC10845550 38317220

[119] Denisenko E, Guo BB, Jones M, Hou R, de Kock L, Lassmann T, et al. Systematic assessment of tissue dissociation and storage biases in single-cell and single-nucleus RNA-seq workflows. Genome Biol. 2020;2(21):130. 10.1186/s13059-020-02048-6PubMedPMID:32487174;PubMedCentralPMCID:PMC7265231. PMC7265231 32487174

[120] Rabilloud T, Lescuyer P. The proteomic to biology inference, a frequently overlooked concern in the interpretation of proteomic data: a plea for functional validation. Proteomics. 2014;14(2–3):157–61. 10.1002/pmic.201300413. 24273051

[121] Anh NK, Thu NQ, Tien NTN, Long NP, Nguyen HT. Advancements in mass spectrometry-based targeted metabolomics and lipidomics: implications for clinical research. Molecules. 2024;29(24):5934. 10.3390/molecules29245934. PMC11677340 39770023

[122] Sopic M, Vilne B, Gerdts E, Trindade F, Uchida S, Khatib S, et al. Multiomics tools for improved atherosclerotic cardiovascular disease management. Trends Mol Med. 2023;29(12):983–95. 10.1016/j.molmed.2023.09.004. 37806854

[123] Lin M, Guo J, Gu Z, Tang W, Tao H, You S, et al. Machine learning and multi-omics integration: advancing cardiovascular translational research and clinical practice. J Transl Med. 2025;23(1):388. 10.1186/s12967-025-06425-2. PMC11966820 40176068

[124] Maringanti R, van Dijk CGM, Meijer EM, Brandt MM, Li M, Tiggeloven VPC, et al. Atherosclerosis on a Chip: A 3-dimensional microfluidic model of early arterial events in human plaques. Arterioscler Thromb Vasc Biol. 2024;44(12):2453–72. 10.1161/ATVBAHA.124.321332. 39297206

[125] Kardassis D, Vindis C, Stancu CS, Toma L, Gafencu AV, Georgescu A, et al. Unravelling molecular mechanisms in atherosclerosis using cellular models and omics technologies. Vascul Pharmacol. 2025;158:107452. 10.1016/j.vph.2024.107452. 39667548

